# Dose–response association and predictive value of plasma aldosterone concentration for cardiometabolic multimorbidity in patients with hypertension: a retrospective cohort study

**DOI:** 10.3389/fendo.2026.1965927

**Published:** 2026-10-16

**Authors:** Zhiqiang Zhang, Nanfang Li, Qing Zhu, Junli Hu, Jing Hong, Wenbo Yang, Pan Zhou, Huimin Ma, Rui Ma, Ting Wu

**Affiliations:** 1 Hypertension Center of People’s Hospital of Xinjiang Uygur Autonomous Region, Urumqi, Xinjiang, China; 2 Xinjiang Hypertension Institute, Urumqi, Xinjiang, China; 3 National Health Commission (NHC) Key Laboratory of Hypertension Clinical Research, Urumqi, Xinjiang, China; 4 Key Laboratory of Xinjiang Uygur Autonomous Region “Hypertension Research Laboratory”, Urumqi, Xinjiang, China; 5 Xinjiang Clinical Medical Research Center for Hypertension (Cardio-Cerebrovascular) Diseases, Urumqi, Xinjiang, China

**Keywords:** cardiometabolic multimorbidity, dose–response relationship, hypertension, plasma aldosterone concentration, predictive value

## Abstract

**Background:**

The purpose of this study was to investigate the relationship between cardiometabolic multimorbidity (CMM) and plasma aldosterone concentration (PAC) among patients with hypertension, to characterize the potential dose–response pattern, and to assess whether the inclusion of PAC into a conventional risk model improves risk reclassification and clinical utility.

**Methods:**

Between January 2010 and January 2026, 7,677 hospitalized hypertensive patients without CMM at baseline were included in this retrospective cohort analysis. The follow-up period was 3.52 years on average. Multivariable Cox proportional hazards regression and restricted cubic spline (RCS) analyses were performed to examine the association between PAC and the risk of incident CMM. The incremental predictive value of PAC was evaluated for 3-year risk prediction using time-dependent ROC analysis with inverse probability of censoring weighting (IPCW), survival-based net reclassification improvement (NRI), integrated discrimination improvement (IDI), calibration assessment, and decision curve analysis (DCA). Based on the potential interaction observed in the subgroup analysis, RCS analyses stratified by serum potassium level and a bivariate penalized spline model were used to characterize the joint nonlinear associations of serum potassium and PAC with the risk of CMM. The Boruta algorithm was additionally applied for feature selection and assessment of variable importance.

**Results:**

During follow-up, 1,949 incident cases of CMM were documented. Patients in the highest quartile of baseline PAC (Q4) had a considerably greater cumulative likelihood of CMM than those in the other quartiles, according to Kaplan–Meier analysis (log-rank *P* < 0.05). In the multivariable Cox regression analysis, each 1-ng/dL increment in PAC was associated with a 2% higher hazard of incident CMM (hazard ratio [HR], 1.02; 95% confidence interval [CI], 1.02–1.03). Compared with the lowest quartile (Q1), the adjusted HRs were 1.19 (95% CI, 1.04–1.36) for Q2, 1.32 (95% CI, 1.16–1.52) for Q3, and 1.65 (95% CI, 1.45–1.87) for Q4, with a significant trend across quartiles (*P* for trend<0.001). RCS analysis demonstrated a significant nonlinear association between PAC and the risk of CMM (*P* for nonlinearity < 0.001), identifying an estimated inflection point at approximately 15.34 ng/dL, above which the risk curve showed an upward trend. Subgroup analyses indicated that the PAC–CMM association may differ by serum potassium status (*P* for interaction < 0.05). After PAC was added to the baseline prediction model, the time-dependent AUC increased from 0.678 to 0.735 (*P* < 0.001), with a net reclassification improvement of 0.372 and an integrated discrimination improvement of 0.038 (*P* < 0.05). DCA suggested that incorporating PAC may provide additional estimated net benefit across clinically relevant threshold probabilities. The Boruta feature-selection algorithm identified PAC as an important predictor, and the overall findings remained consistent across subgroup and sensitivity analyses.

**Conclusion:**

The possibility of a CMM incident was positively correlated with PAC in a nonlinear fashion, and this correlation differed depending on serum potassium levels. Incorporating PAC into a conventional risk model was associated with improved predictive performance and greater net clinical benefit, suggesting that PAC may provide additional prognostic information for cardiometabolic risk stratification among patients with hypertension.

## Highlights

- This study is the first to systematically investigate the association between plasma aldosterone concentration and incident cardiometabolic multimorbidity in patients with hypertension.
- Higher plasma aldosterone concentrations were independently associated with an increased risk of incident cardiometabolic multimorbidity, with a significant nonlinear exposure–response relationship.
- A plasma aldosterone concentration of approximately 15.34 ng/dL was identified as a potential risk-related inflection point, and incorporating plasma aldosterone concentration into a conventional prediction model significantly improved risk discrimination and reclassification.

## 1 Introduction

The term cardiometabolic multimorbidity (CMM) describes individuals affected by at least two major cardiometabolic disorders, such as coronary heart disease, stroke, or type 2 diabetes mellitus. The coexistence of these conditions contributes substantially to mortality and disability worldwide and imposes an increasing burden on healthcare systems (1, 2). CMM is associated with greater all-cause mortality than the presence of a single cardiometabolic disease (CMD). This excess mortality highlights the need for early recognition of vulnerable patients and coordinated management of coexisting cardiometabolic conditions (1, 3, 4). Hypertension is highly prevalent and represents an important modifiable factor associated with CMD. Its long-term effects on the cardiovascular and renal systems may be linked to a pathophysiological milieu in which CMM tend to coexist (5–7). Nevertheless, despite continued advances in antihypertensive treatment, patients with hypertension remain exposed to substantial residual cardiovascular risk, suggesting that factors beyond blood pressure itself may be associated with progression toward CMM (6, 8).

Plasma aldosterone concentration (PAC) is a major terminal effector of the renin–angiotensin–aldosterone system (RAAS), and its biological actions extend beyond the classical regulation of sodium retention and potassium excretion. Experimental and clinical evidence suggests that aldosterone may be involved in endothelial dysfunction, oxidative stress, inflammation, and myocardial fibrosis through both mineralocorticoid receptor (MR)-dependent and MR-independent pathways, thereby contributing to cardiovascular target-organ injury (9, 10). Patients with primary aldosteronism (PA) have been reported to experience higher rates of cardiovascular events, including stroke, coronary heart disease, and heart failure (11). Importantly, cardiovascular abnormalities associated with PAC excess may not be confined to patients with PA (12, 13). Studies conducted in populations without PA have shown that, even after PA is excluded, higher PAC remains continuously associated with adverse cardiovascular outcomes (14–16). Collectively, these findings suggest that PAC may be linked to the development and coexistence of cardiometabolic conditions in patients with hypertension through pathways involving MR activation, vascular injury, myocardial remodeling, and disturbances in glucose and lipid metabolism.

However, evidence regarding the association between PAC and CMM remains limited. Previous studies have largely focused on patients with PA or on individual outcomes such as coronary heart disease, stroke, and heart failure. The continuous association between PAC and incident CMM, including its potential nonlinear pattern, has not been systematically characterized. Furthermore, it remains uncertain whether PAC provides additional predictive information beyond established risk factors for CMM among patients with hypertension. Accordingly, using a retrospective cohort of patients with hypertension, this study aimed to evaluate the association between PAC and incident CMM and to assess the incremental predictive value of PAC. The specific objectives were to: (1) characterize the continuous pattern of the association between PAC and the risk of incident CMM; (2) examine whether this association was nonlinear and identify a potential risk-related inflection point; and (3) determine whether adding PAC to a model containing conventional risk factors improved the prediction of CMM. These findings may provide additional epidemiological evidence for cardiometabolic risk stratification in patients with hypertension and inform future investigations of RAAS-related preventive and therapeutic strategies.

## 2 Materials and methods

### 2.1 Patient selection

This retrospective cohort study included patients with hypertension who were admitted to the People’s Hospital of Xinjiang Uygur Autonomous Region between January 2010 and January 2026. Patients were excluded if they met any of the following criteria: (1) age <18 years; (2) missing data on PAC or CMM; (3) a documented diagnosis of diabetes, heart disease, or stroke at baseline; (4) loss to follow-up or a follow-up duration of <6 months; (5) a history of severe hepatic or renal dysfunction, hyperthyroidism, primary aldosteronism, or an acute cardiovascular event within the preceding 6 months; or (6) previous use of a mineralocorticoid receptor antagonist. After application of these criteria, 7,677 patients were included in the final analysis. The participant selection process is presented in **Figure 1**. The study protocol was approved by the Ethics Committee of the People’s Hospital of Xinjiang Uygur Autonomous Region (Approval Nos. KY202080905 and KY2026011506). The requirement for informed consent was waived because the study involved retrospective collection and analysis of existing clinical data. The study was reported in accordance with the Strengthening the Reporting of Observational Studies in Epidemiology (STROBE) recommendations.

**Figure 1:**
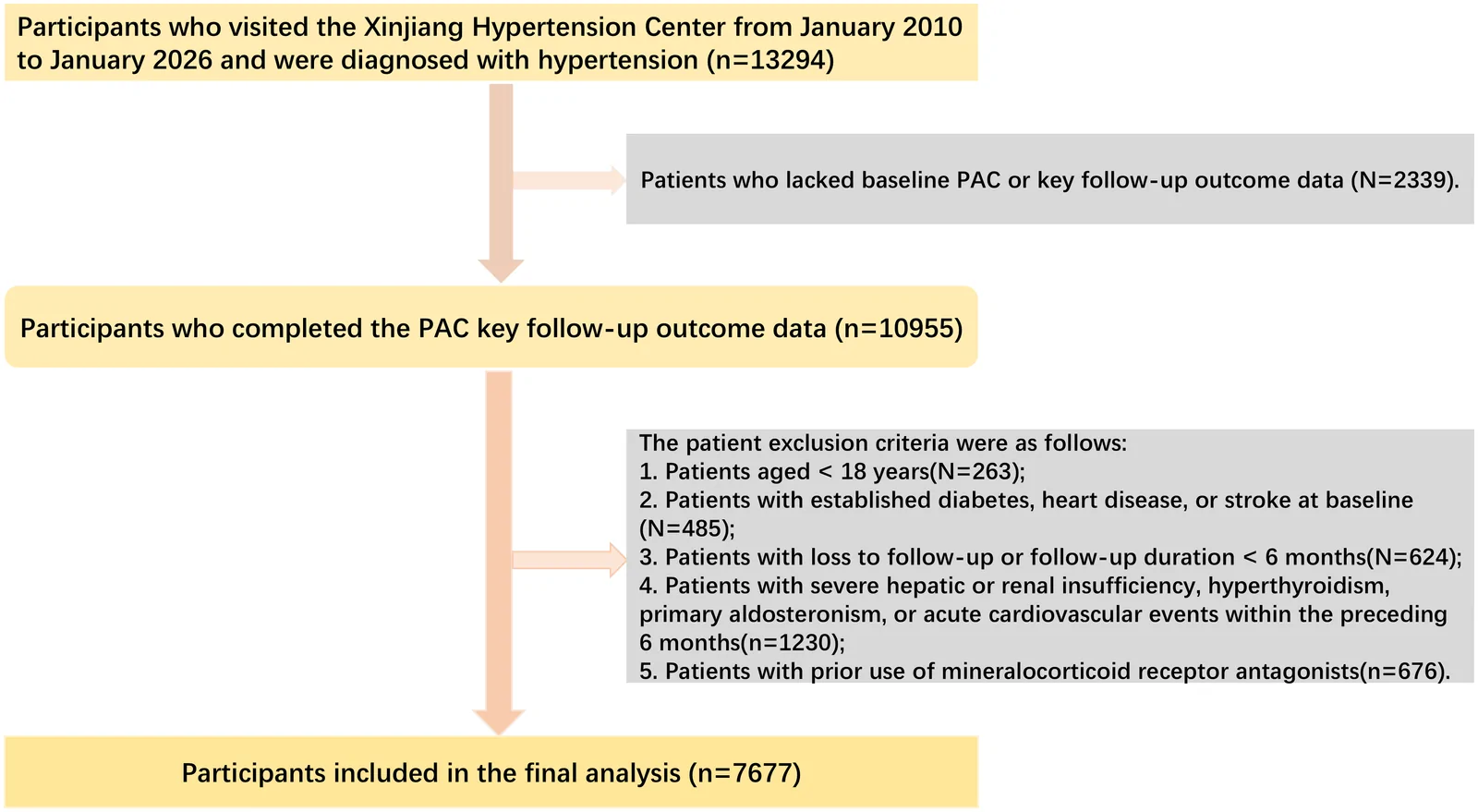
Flowchart for selection of study populations.

### 2.2 Data collection and definitions

Baseline demographic characteristics, clinical information, laboratory measurements, lifestyle factors, medical history, and medication use were obtained from electronic medical records. Clinical variables collected at hospital admission included age, sex, height, weight, body mass index (BMI), blood pressure (BP), and waist circumference (WC). Smoking status was categorized as non-smokers or current smokers, while alcohol consumption status was classified as non-drinkers or current drinkers. Detailed information regarding the definitions and measurement procedures of lifestyle variables is provided in the **Supplementary Materials**. After an overnight fast, venous blood samples were collected from all participants for biochemical and hormonal assessments. The following laboratory parameters were measured: glycated hemoglobin (HbA1c), fasting plasma glucose (FPG), total cholesterol (TC), triglycerides (TG), high-density lipoprotein cholesterol (HDL-C), low-density lipoprotein cholesterol (LDL-C), alanine aminotransferase (ALT), aspartate aminotransferase (AST), blood urea nitrogen (BUN), serum creatinine (Scr), uric acid (UA), parathyroid hormone (PTH), thyroid-stimulating hormone (TSH), Plasma Renin Activity (PRA), Aldosterone-to-Renin Ratio (ARR), serum potassium, serum sodium, and serum calcium. All biochemical parameters were measured using an automated analyzer (7600-010; Hitachi, Tokyo, Japan) according to the manufacturer’s instructions. Hormonal assessments were performed in accordance with current clinical guidelines and our previous studies (6, 17, 18). PAC was quantified using a radioimmunoassay method (DSL-8600; Diagnostic Systems Laboratories, Webster, TX, USA). ARR is calculated by measuring PAC and PRA. Detailed procedures for laboratory measurements and quality control are described in the **Supplementary Materials**. The definitions of comorbid conditions, including hyperlipidemia and other cardiometabolic disorders, were established according to current diagnostic criteria. Detailed diagnostic definitions and classification procedures are provided in the **Supplementary Materials**.

### 2.3 Outcome assessment

The primary outcome was incident CMM during follow-up. CMM was defined as the coexistence of at least two cardiometabolic diseases in the same participant, including diabetes, heart disease, and stroke (19, 20). A participant was considered to have developed CMM on the date when the second of these conditions was first diagnosed (21). Heart disease and stroke were identified on the basis of self-reported physician diagnoses and/or International Classification of Diseases (ICD-10) codes recorded in the electronic medical records. Diabetes was ascertained using self-reported physician diagnosis, use of glucose-lowering medications, FPG, HbA1c, and/or relevant ICD-10 codes. Because all participants were free of diabetes, heart disease, and stroke at baseline, incident CMM was defined as the first occurrence of at least two of these three conditions during follow-up. For participants who developed cardiometabolic diseases at different follow-up time points, the date of CMM onset was defined as the date on which the second cardiometabolic disease was first diagnosed. (ICD-10) codes are provided in the **Supplementary Materials**.

### 2.4 Statistical analysis

The extent of missing data for all variables included in this study is presented in **Supplementary Figure S1**. Multicollinearity among predictor variables was assessed using the variance inflation factor (VIF) (**Supplementary Figure S2**). Kaplan–Meier survival curves and log-rank tests were used to compare differences in the cumulative incidence of CMM across different PAC categories. The proportional hazards (PH) assumption for Cox proportional hazards models was evaluated using scaled Schoenfeld residuals, incorporating both graphical assessments of residual patterns over follow-up time and formal statistical tests to determine whether the PH assumption was satisfied. To investigate the independent association between PAC and the risk of incident CMM, four progressively adjusted multivariable Cox proportional hazards regression models were constructed. Covariates were selected *a priori* and sequentially incorporated into the models, including sociodemographic characteristics, lifestyle factors, clinical parameters, biochemical measurements, and antihypertensive medication use. Hazard ratios (HRs) and corresponding 95% confidence intervals (CIs) were estimated for each model. Restricted cubic spline (RCS) analyses were performed to examine potential nonlinear dose–response relationships between PAC levels and the risk of CMM. To further explore whether serum potassium levels modified the association between PAC and CMM risk, a bivariate penalized spline model was constructed to characterize variations in log hazard ratios (log-HRs) associated with PAC across different serum potassium levels. In addition, interaction terms between PAC and serum potassium levels were incorporated into multivariable Cox regression models to quantitatively assess potential statistical interactions. A series of subgroup analyses and sensitivity analyses were performed to evaluate the consistency and robustness of the association between PAC and CMM risk across clinically relevant subgroups. To assess the incremental predictive value of PAC for CMM risk prediction, the predictive performance of a baseline model was compared with that of an extended model incorporating PAC. The baseline model included age, sex, BMI, SBP, DBP, current smoking status, current alcohol consumption, TG, HDL-C, HbA1c, LVEF, FPG, and antihypertensive medication use, including calcium channel blockers, β-blockers, angiotensin-converting enzyme inhibitors/angiotensin II receptor blockers (ACEIs/ARBs), and diuretics. All prediction analyses were prespecified using a 3-year prediction horizon, which was selected based on the median follow-up duration of the cohort. Because the occurrence of CMM was analyzed as a time-to-event outcome with variable follow-up durations and right censoring, time-dependent receiver operating characteristic (ROC) analysis incorporating inverse probability of censoring weighting (IPCW) was performed to evaluate model discrimination at 3 years. The area under the time-dependent ROC curve (AUC) and corresponding 95% confidence intervals (CIs) were calculated, and differences between models were assessed. Internal validation was performed using 1,000 bootstrap resampling iterations to obtain optimism-corrected estimates of model performance. The incremental predictive value of PAC was further assessed using continuous survival-based net reclassification improvement (NRI) and integrated discrimination improvement (IDI), based on predicted 3-year absolute risks derived from Cox proportional hazards models while accounting for censoring. Model calibration was evaluated by comparing predicted and observed 3-year risks using calibration plots. Furthermore, decision curve analysis (DCA) was conducted using predicted 3-year risks to estimate the potential net benefit of incorporating PAC into the baseline model. Additional details regarding statistical procedures are provided in the **Supplementary Material**.

Statistical analyses were conducted using R (version 4.5.2). Two-sided P values below 0.05 were regarded as statistically significant.

## 3 Results

### 3.1 Baseline characteristics of the study population

The final analysis comprised 7,677 participants who were free of CMM at baseline. Baseline characteristics of the cohort are presented in **Table 1**. The participants had a mean age of 52.31 ± 11.59 years, and men accounted for 57.60% of the study population.

**Table 1:** Characteristics of the study population based on PAC quartiles.

Characteristic	Overall	Q1	Q2	Q3	Q4	p value
sample size, n	7677	1917	1917	1922	1921	
Age, years	52.31± 11.59	53.87± 12.06	53.20± 11.42	51.79± 11.13	50.41± 11.45	<0.001
Sex, n (%)						0.002
Male	4422 (57.60%)	1174 (61.24%)	1074 (56.03%)	1100 (57.23%)	1074 (55.91%)	
Female	3255 (42.40%)	743 (38.76%)	843 (43.97%)	822 (42.77%)	847 (44.09%)	
BMI, kg/m^2^	27.22 ± 3.88	27.03± 3.96	27.21± 4.02	27.38± 3.70	27.25± 3.81	0.043
SBP, mmHg	144.52± 19.81	143.72± 19.78	144.84± 19.72	144.33± 19.51	145.19± 20.19	0.112
DBP, mmHg	88.13± 14.36	86.55± 13.97	87.37± 13.98	88.69± 13.97	89.93± 15.24	<0.001
Current smoking, n (%)	2584 (33.66%)	754 (39.33%)	625 (32.60%)	612 (31.84%)	593 (30.87%)	<0.001
Current drinking, n (%)	2224 (28.97%)	614 (32.03%)	537 (28.01%)	538 (27.99%)	535 (27.85%)	0.009
Laboratory results						
FPG, mmol/L	5.17± 1.39	5.19± 1.42	5.10± 1.31	5.19± 1.41	5.18± 1.42	0.173
HbA1c, %	6.04± 0.98	6.12± 1.03	6.04 ± 0.95	6.04± 1.01	5.96± 0.94	<0.001
TG, mmol/L	1.84± 1.44	1.81± 1.39	1.83± 1.57	1.87± 1.46	1.83± 1.35	0.590
HDL-C, mmol/L	1.09± 0.28	1.10± 0.29	1.09± 0.28	1.09± 0.28	1.09± 0.28	0.758
ALT, IU/L	22.74 (16.00-33.00)	22.00 (15.42-31.26)	22.20 (16.00-33.00)	23.00 (16.70-33.45)	23.00 (16.40-33.50)	0.035
AST, IU/L	19.40 (16.00-24.00)	19.00 (16.00-23.63)	19.00 (16.00-24.00)	19.62 (16.00-24.00)	20.00 (16.00-24.10)	0.333
BUN, mmol/L	5.31 ± 1.70	5.23± 1.51	5.23± 1.58	5.26± 1.54	5.51± 2.08	<0.001
Scr, umol/L	65.90 (55.80-77.10)	65.00 (55.80-75.80)	64.70 (54.50-75.07)	66.00 (55.91-77.00)	67.80 (57.10-81.00)	<0.001
Serum potassium, mmol/L	3.91± 0.41	3.92 ± 0.38	3.91 ± 0.40	3.92± 0.40	3.91± 0.47	0.718
Serum calcium, mmol/L	2.22± 0.30	2.23± 0.28	2.21± 0.31	2.23± 0.26	2.22± 0.34	0.120
Serum sodium, mmol/L	141.01 ± 2.43	141.07 ± 2.49	141.09 ± 2.37	141.16 ± 2.34	140.71 ± 2.51	<0.001
TSH, µIU/mL	2.02 (1.38-3.03)	2.01 (1.34-2.97)	2.07 (1.43-3.10)	2.01 (1.38-3.02)	2.02 (1.38-3.03)	0.250
PTH, PG/mL	53.70 (39.50-72.00)	52.90 (38.20-69.20)	53.20 (39.80-69.70)	53.25 (39.42-72.80)	56.40 (40.50-76.60)	<0.001
LVEF	61.04± 4.08	60.93± 4.04	61.10± 4.18	61.05± 3.74	61.07± 4.33	0.578
PAC, ng/dL	17.38± 8.33	10.18± 1.70	13.40± 0.86	17.41± 1.55	28.51± 8.92	<0.001
PRA ng/mL/h	1.95 (0.74-3.26)	1.68 (0.56-2.85)	1.73 (0.65-2.83)	2.03 (0.84-3.28)	2.38 (1.13-3.89)	<0.001
ARR	8.75 (4.93-20.48)	6.16 (3.43-17.39)	7.80 (4.74-20.16)	8.65 (5.46-20.68)	11.39 (6.90-23.55)	<0.001
Comorbidities (%)						
Hyperlipidaemia	699 (9.11%)	163 (8.50%)	179 (9.34%)	189 (9.83%)	168 (8.75%)	0.476
Medication use, n (%)						
Calcium channel blockers	6171 (80.38%)	1464 (76.37%)	1505 (78.51%)	1558 (81.06%)	1644 (85.58%)	<0.001
Beta-blockers	2112 (27.51%)	542 (28.27%)	491 (25.61%)	511 (26.59%)	568 (29.57%)	0.030
ACEIs/ARBs	4736 (61.69%)	1263 (65.88%)	1168 (60.93%)	1164 (60.56%)	1141 (59.40%)	<0.001
Diuretics	1166 (15.19%)	251 (13.09%)	278 (14.50%)	294 (15.30%)	343 (17.86%)	<0.001
Lipidloweringagents	2351 (30.62%)	623 (32.50%)	570 (29.73%)	595 (30.96%)	563 (29.31%)	0.135

Data are presented as mean  ±  standard deviation, median (interquartile range), or as numbers and percentages. BMI, body mass index; SBP, systolic blood pressure; DBP, diastolic blood pressure; FPG: fasting plasma glucose; HbA1c: Glycated Hemoglobin: HDL-C, high-density lipoprotein cholesterol; TG, total triglyceride; AST, aspartate aminotransferase; ALT, alanine aminotransferase; Scr, serum creatinine; TSH, Thyroid - Stimulating Hormone; PTH, parathyroid hormone; LVEF, Left Ventricular Ejection Fraction; ACEIs, angiotensin-converting enzyme inhibitors; ARBs, angiotensin receptor blockers; PAC, Plasma Aldosterone Concentration; PRA plasma renin activity, ARR aldosterone–renin ratio; PTH, Parathyroid Hormone; UA, Uric Acid; BUN, Blood Urea Nitrogen; Scr, Serum Creatinine; P, Probability.

The mean PAC was 17.38 ± 8.33 ng/dL. When participants were categorized according to PAC quartiles, several baseline characteristics differed across the groups. Compared with those in the lowest quartile (Q1), participants in the higher PAC quartiles (Q2–Q4) were generally younger, included a greater proportion of women, and had higher BMI and diastolic blood pressure. In contrast, the proportions of current smokers and drinkers were lower in the higher quartiles (*P* < 0.05). Systolic blood pressure did not differ significantly across the PAC quartiles (*P* = 0.112). Regarding laboratory measurements, participants in Q2–Q4 had lower glycated hemoglobin levels and higher concentrations of blood urea nitrogen, serum creatinine, and parathyroid hormone, together with lower serum sodium levels (*P* < 0.001). PRA and ARR increased progressively across PAC quartiles (*P* < 0.001). Alanine aminotransferase levels also differed significantly among the groups (*P* = 0.035). FPG, TG, HDL-C, AST, serum potassium, serum calcium, TSH, LVEF, and the prevalence of hyperlipidemia were comparable across PAC quartiles (*P* > 0.05).

### 3.2 Association between PAC and incident CMM

1,949 incident cases of CMM were recorded over a median follow-up of 3.52 years (interquartile range, 1.98–5.57 years). Cumulative CMM incidence rose with increasing baseline PAC categories (**Figure 2**). Consistent with this pattern, Kaplan–Meier curves demonstrated the greatest cumulative probability of CMM in participants in the highest PAC quartile (Q4) compared with the remaining quartiles during follow-up (log-rank *P* < 0.05; **Figure 3**). Assessment using Schoenfeld residuals showed that the proportional hazards assumption was satisfied for all Cox models (**Supplementary Figure S3**). Multivariable Cox regression results are provided in **Table 2**. After full covariate adjustment, a 1-ng/dL increase in PAC corresponded to an HR of 1.02 for incident CMM (95% CI, 1.02–1.03), whereas the HR per 1-standard-deviation increase was 1.22 (95% CI, 1.18–1.26). When PAC was dichotomized, the Q3–Q4 group showed a higher hazard of CMM than the Q1–Q2 group (HR, 1.36; 95% CI, 1.25–1.50). The association persisted in the fully adjusted model (Model 4), and a graded pattern was observed across PAC categories (P for trend < 0.001). Using Q1 as the reference, the adjusted HRs were 1.19 (95% CI, 1.04–1.36) for Q2, 1.32 (95% CI, 1.16–1.52) for Q3, and 1.65 (95% CI, 1.45–1.87) for Q4. Restricted cubic spline analysis indicated a nonlinear relationship between PAC and CMM (*P* for nonlinearity < 0.001; **Figure 4**), with the estimated hazard showing an upward trend across increasing PAC levels beyond 15.34 ng/dL. In the two-stage analysis, participants with PAC >15.34 ng/dL had an estimated CMM risk 1.01 times that of those with PAC ≤15.34 ng/dL (**Table 3**).

**Figure 2:**
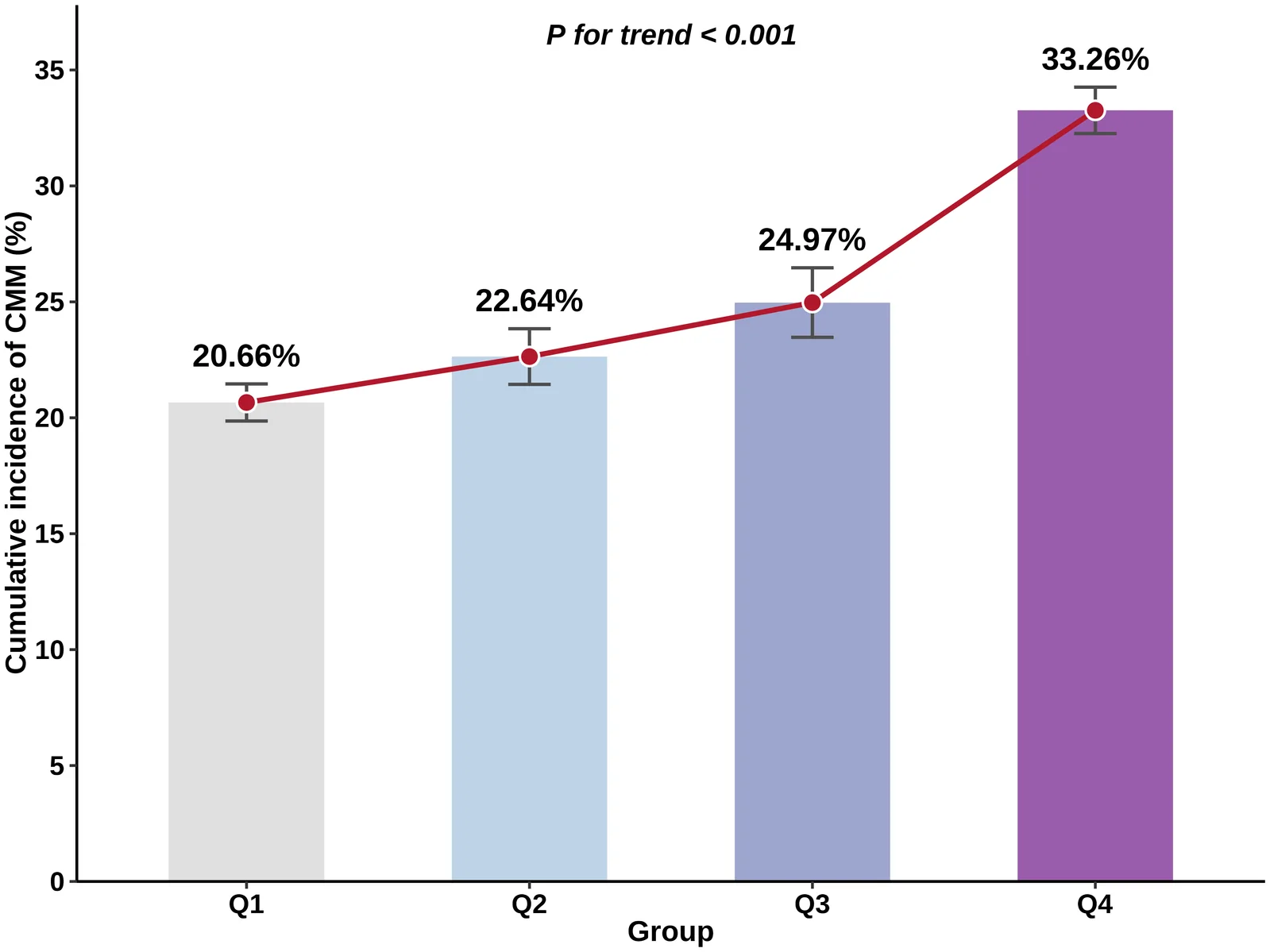
Cumulative incidence of CMM stratified by PAC quartiles.

**Figure 3:**
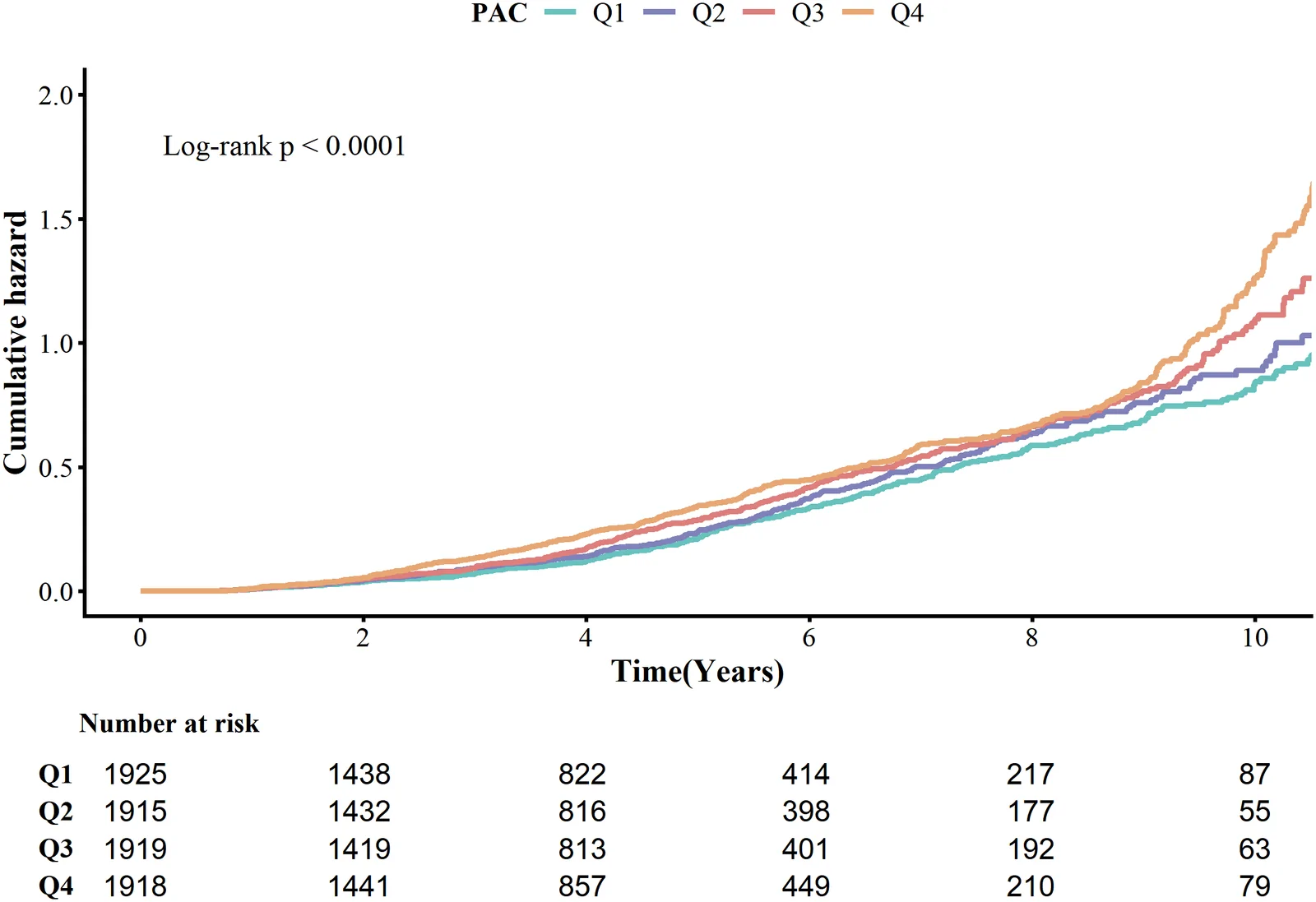
Cumulative incidence of CMM According to PAC quartiles.

**Table 2:** Association of PAC with CMM.

Exposure	Model 1	Model 2	Model 3	Model 4	Bootstraping^a^
	HR (95% CI) P	HR (95% CI) P	HR (95% CI) P	HR (95% CI) P	HR (95% CI) P
CMM					
PAC (per 1-ng/dL increase)	1.02 (1.02, 1.03) <0.001	1.02 (1.02, 1.03) <0.001	1.02 (1.02, 1.03) <0.001	1.02 (1.02, 1.03) <0.001	1.03 (1.02, 1.03) <0.001
PAC (per 1 SD increase)	1.21 (1.17, 1.24) <0.001	1.22 (1.19, 1.26) <0.001	1.22 (1.19, 1.26) <0.001	1.22 (1.18–1.26) <0.001	1.23 (1.19, 1.29) <0.001
Quartiles of PAC					
Q1	Reference	Reference	Reference	Reference	Reference
Q2	1.16 (1.01, 1.33) <0.001	1.18 (1.03, 1.35) <0.001	1.17 (1.02, 1.35) <0.001	1.19 (1.04, 1.36) <0.001	1.19 (1.03, 1.37) <0.001
Q3	1.28 (1.12, 1.46) <0.001	1.34 (1.17, 1.53) <0.001	1.33 (1.16, 1.52) <0.001	1.32 (1.16, 1.52) <0.001	1.32 (1.16, 1.52) <0.001
Q4	1.55 (1.37, 1.76) <0.001	1.65 (1.45, 1.87) <0.001	1.64 (1.45, 1.86) <0.001	1.65 (1.45, 1.87) <0.001	1.65 (1.43, 1.88) <0.001
Q1-Q2	Reference	Reference	Reference	Reference	Reference
Q3-Q4	1.32 (1.21, 1.45) <0.001	1.38 (1.26, 1.51) <0.001	1.38 (1.26, 1.52) <0.001	1.36 (1.25, 1.50) <0.001	1.38 (1.26, 1.51) <0.001
P for trend	<0.001	<0.001	<0.001	<0.001	<0.001

Model 1: No covariates were adjusted.

Model 2: Adjusted for SEX, AGE, BMI, Current smoking, Current drinking.

Model 3: Adjusted for SEX, AGE, BMI, Current smoking, Current drinking, SBP, DBP, ALT, AST, TG, HDL-C, LVEF, Serum potassium, Serum calcium, Serum sodium.

Model 4: Adjusted for SEX, AGE, BMI, Current smoking, Current drinking, SBP, DBP, ALT, AST, TG, HDL-C, LVEF, Serum potassium, Serum calcium, Serum sodium, Scr, ACEIs/ARBs; Calcium channel blockers, Beta-blockers, Diuretics.

^a^ Bootstrapping was conducted with 1000 bootstrap samples via Adjusted Model 4.

ACEIs, angiotensin-converting enzyme inhibitors; ARBs, angiotensin receptor blockers; PAC, Plasma Aldosterone Concentration; HR, Hazard Ratio; CI, Confidence Interval; P, Probability.

Other abbreviations, see **Table 1**.

**Figure 4:**
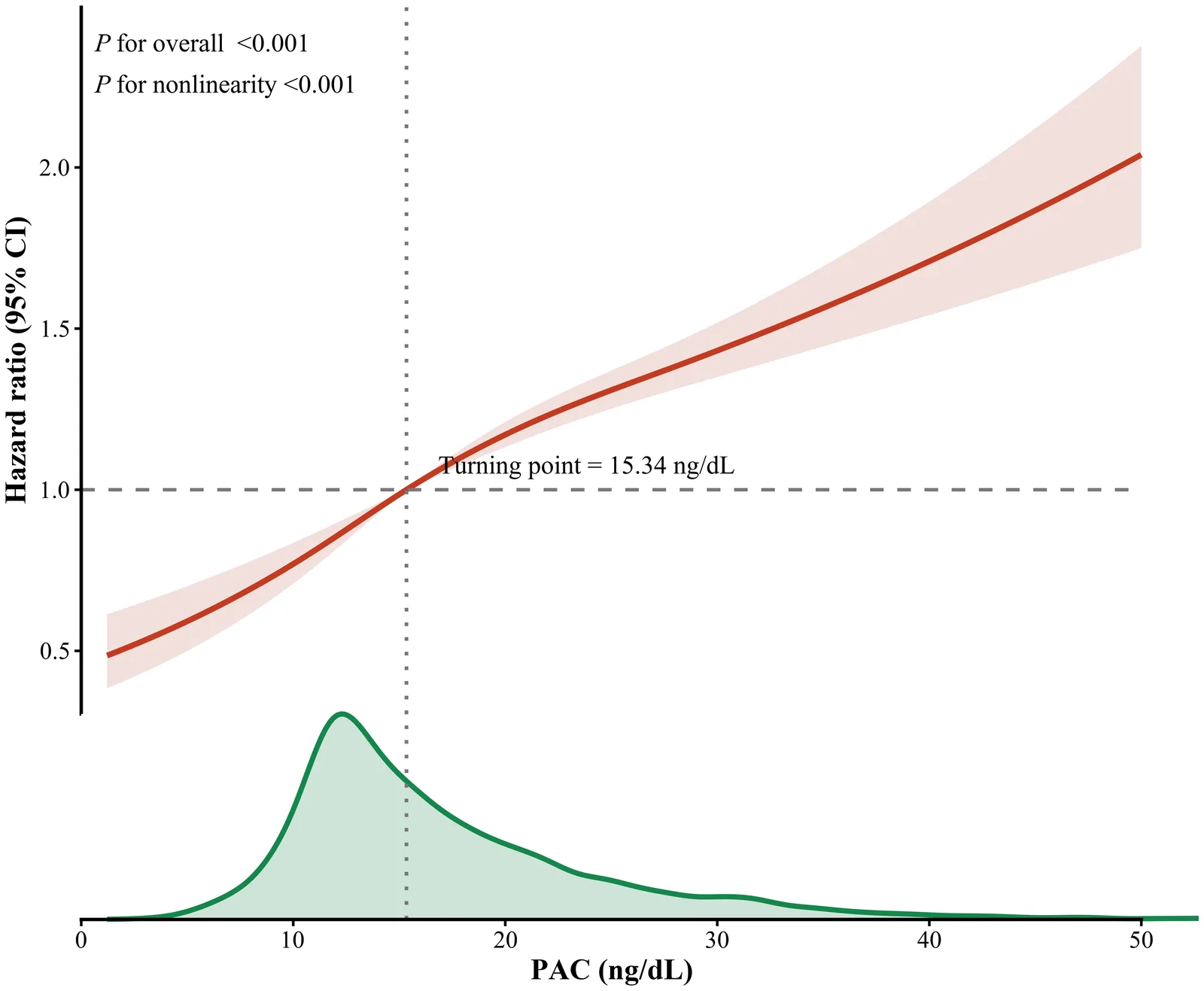
Restricted cubic splines for the association between PAC and CMM.

**Table 3:** Two-stage comparative analysis of the relationship between PAC and CMM based on the RCS turning points.

Exposure	Model 1	Model 2	Model 3	Model 4
	HR (95% CI) P	HR (95% CI) P	HR (95% CI) P	HR (95% CI) P
CMM				
Turning point (ng/dL)	15.34	15.34	15.34	15.34
<= 15.34	Reference	Reference	Reference	Reference
> 15.34	1.01 (1.00, 1.02) <0.001	1.01 (1.01, 1.02) <0.001	1.06 (1.03, 1.08) <0.001	1.01 (1.01, 1.02) <0.001

Model 1: No covariates were adjusted.

Model 2: Adjusted for SEX, AGE, BMI, Current smoking, Current drinking.

Model 3: Adjusted for SEX, AGE, BMI, Current smoking, Current drinking, SBP, DBP, ALT, AST, TG, HDL-C, LVEF, Serum potassium, Serum calcium, Serum sodium.

Model 4: Adjusted for SEX, AGE, BMI, Current smoking, Current drinking, SBP, DBP, ALT, AST, TG, HDL-C, LVEF, Serum potassium, Serum calcium, Serum sodium, Scr, ACEIs/ARBs; Calcium channel blockers, Beta-blockers, Diuretics.

^a^ Bootstrapping was conducted with 1000 bootstrap samples via Adjusted Model 4.

ACEIs, angiotensin-converting enzyme inhibitors; ARBs, angiotensin receptor blockers; PAC, Plasma Aldosterone Concentration; HR, Hazard Ratio; CI, Confidence Interval; P, Probability. Other abbreviations, see **Table 1**.

### 3.3 Subgroup analyses

Given that the cohort comprised only individuals with hypertension, potential heterogeneity related to baseline comorbidities and medication use was further examined. Subgroup analyses were conducted according to sex, age, BMI, PRA, ARR, smoking status, drinking consumption status, serum potassium, and hyperlipidemia. The association of higher PAC with incident CMM was broadly similar across these prespecified strata (**Table 4**). We also examined the results within subgroups defined by antihypertensive treatment. Comparable estimates were obtained across medication categories, with no substantial departure from the main analysis, suggesting that the observed relationship was largely unchanged after stratification by these factors (**Supplementary Table S1**).

**Table 4:** Association of PAC with CMM across subgroups.

CMM			
PAC (per 1- SD increase)	P value	HR (95% CI)	P for interaction
Overall	<0.001	1.21 (1.17, 1.24)	
Age			0.589
<60	<0.001	1.22 (1.17, 1.26)	
>=60	<0.001	1.20 (1.13, 1.27)	
Sex			0.349
Male	<0.001	1.23 (1.17, 1.28)	
Female	<0.001	1.19 (1.14 to 1.24)	
BMI (kg/m2)			0.369
<25	<0.001	1.23 (1.16, 1.30)	
>=25	<0.001	1.19 (1.15, 1.24)	
Current smoking			0.087
No	<0.001	1.19 (1.15, 1.23)	
Yes	<0.001	1.26 (1.19, 1.34)	
Current drinking			0.310
No	<0.001	1.19 (1.15, 1.24)	
Yes	<0.001	1.24 (1.17, 1.32)	
Serum potassium (mmol/L)			**0.035**
<3.5	0.029	1.09 (1.01, 1.18)	
>=3.5	<0.001	1.23 (1.18, 1.27)	
Hyperlipidaemia			0.634
No	<0.001	1.21 (1.17 to 1.25)	
Yes	<0.001	1.17 (1.05 to 1.32)	
ARR			0.101
<20	<0.001	1.23 (1.19, 1.28)	
>=20	0.007	1.16 (1.10, 1.23)	
PRA (ng/mL/h)			0.054
<0.5	0.007	1.12 (1.03, 1.21)	
>=0.5	0.007	1.22 (1.18, 1.27)	

PAC, plasma aldosterone concentration; HR, Hazard Ratio; CI, Confidence Interval.

Other abbreviations, see **Table 1**.

### 3.4 Joint nonlinear interaction between PAC and serum potassium on the risk of CMM

Subgroup analyses indicated broadly similar PAC-related estimates for incident CMM across most prespecified strata. A potential difference emerged after stratification by serum potassium. In participants with potassium ≥3.5 mmol/L, the HR per 1-standard-deviation increment in PAC was 1.23 (95% confidence interval [CI], 1.18–1.27), compared with 1.09 (95% CI, 1.01–1.18) in those with potassium <3.5 mmol/L (*P* for interaction = 0.035; **Table 4**). To further examine the shape of the PAC–CMM relationship according to potassium status, restricted cubic spline analyses were conducted separately within each potassium category. The estimated hazard of CMM rose with increasing PAC in both groups, with a steeper increase in HR observed among participants with potassium ≥3.5 mmol/L. This pattern was generally in line with the subgroup results (**Figure 5**). However, when potassium was treated as a continuous variable and its overall nonlinear interaction with PAC was assessed using flexible spline functions, statistical significance was not reached (*P* = 0.244; **Supplementary Figure S4**). Thus, the heterogeneity identified using the 3.5-mmol/L cutoff may partly depend on the choice of threshold. Given the lack of concordant evidence from the continuous nonlinear interaction analysis, this observation should be viewed as preliminary and interpreted cautiously.

**Figure 5:**
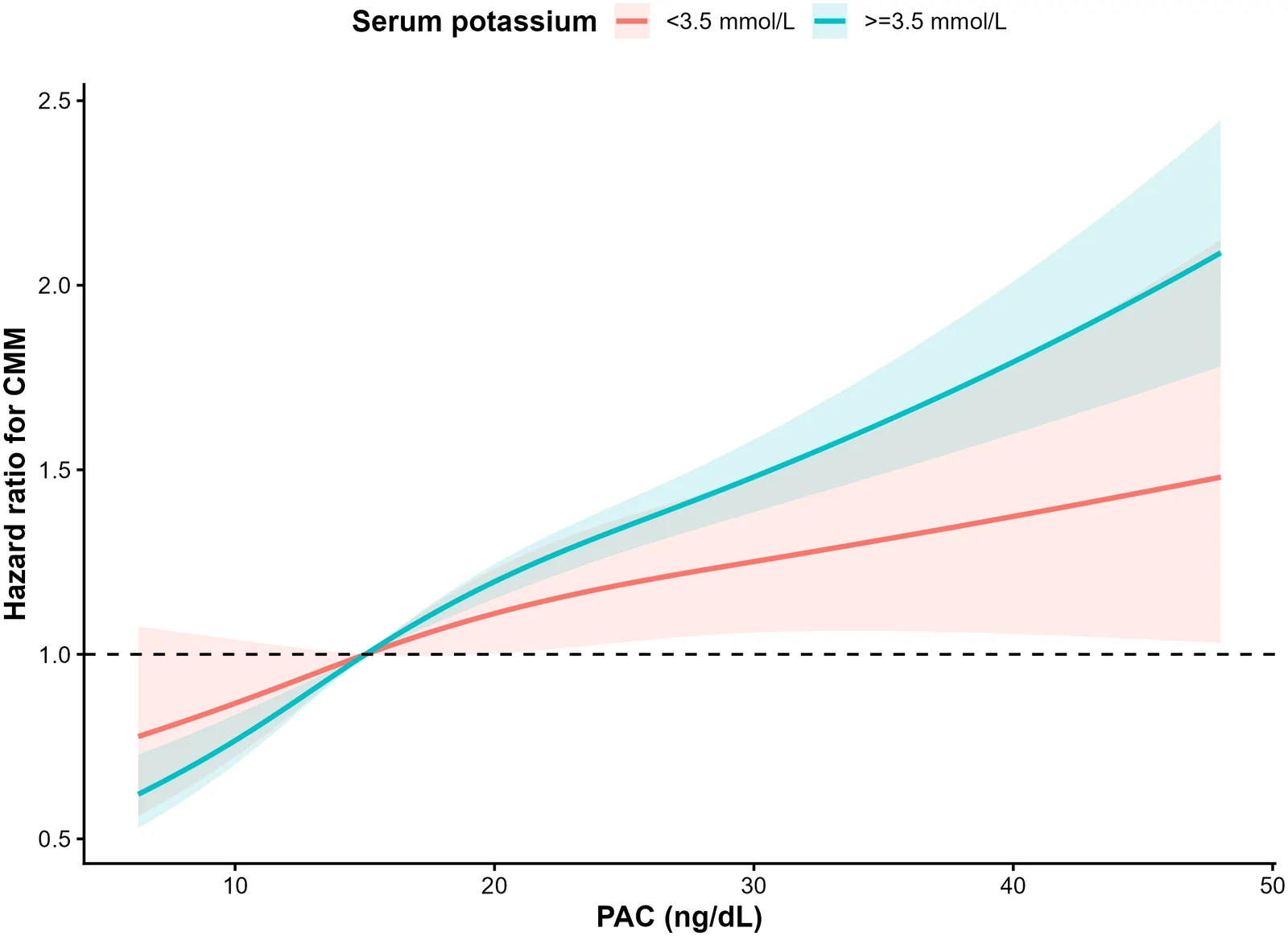
Association between PAC and incident CMM according to serum potassium categories.

### 3.5 Incremental predictive value of PAC

Using a prespecified 3-year prediction horizon, time-dependent ROC analysis showed that incorporating PAC into the baseline model improved discrimination for incident CMM (**Table 5**). Compared with the baseline model, incorporation of PAC was associated with improved discrimination performance. The time-dependent AUC increased from 0.678 (95% CI, 0.664–0.693) for the baseline model to 0.735 (95% CI, 0.722–0.749) for the PAC-integrated model (P for dAUC <0.001), suggesting that adding PAC provided additional discriminatory information for identifying individuals at different risks of developing CMM. After internal validation with 1,000 bootstrap resampling iterations, the optimism-corrected area AUC of the PAC-incorporated model remained 0.730, suggesting that the observed predictive improvement was robust and unlikely to be attributable to model overfitting. In addition, the sensitivity increased from 0.438 in the baseline model to 0.600 after incorporation of PAC, while maintaining a specificity of 0.774, further supporting the enhanced risk discrimination capability of the extended model. ROC curve analysis demonstrated a clear improvement in model discrimination after adding PAC, as reflected by the increased area under the ROC curve (**Figure 6**). Furthermore, incremental prediction analyses showed significant improvements in risk reclassification after incorporating PAC into the baseline model, with a continuous NRI of 0.372 and an IDI of 0.038. The increase in AUC was also statistically significant (*P* < 0.001), suggesting that PAC provided additional prognostic information beyond conventional risk factors (**Table 5**).

**Table 5:** Incremental predictive performance of PAC for 3-year risk prediction of CMM.

Index	3-year time-dependent AUC	95% CI	Optimism-corrected AUC	Optimism estimate	Sensitivity	Specificity	Positive-pv	Negative-pv	P for dAUC	Continuous NRI	IDI
Baseline	0.678	0.664-0.693	0.671	0.008	0.438	0.867	0.529	0.819	Ref.	Ref.	Ref.
PAC + Baseline	0.735	0.722-0.749	0.730	0.006	0.600	0.774	0.475	0.851	< 0.001	0.372	0.038

The basic model included age, sex, BMI, SBP, DBP, current smoking status, current alcohol consumption, TG, HDL-C, HbA1c, LVEF, FPG, and antihypertensive medication use, including calcium channel blockers, β-blockers, ACEIs/ARBs, and diuretics; AUC, area under the time-dependent receiver operating characteristic curve; CI, confidence interval; NRI, continuous net reclassification improvement; IDI, integrated discrimination improvement. Time-dependent ROC analyses were performed using inverse probability of censoring weighting (IPCW) with a prespecified 3-year prediction horizon. Internal validation was conducted using 1,000 bootstrap resampling iterations, and optimism-corrected AUC estimates were reported.

**Figure 6:**
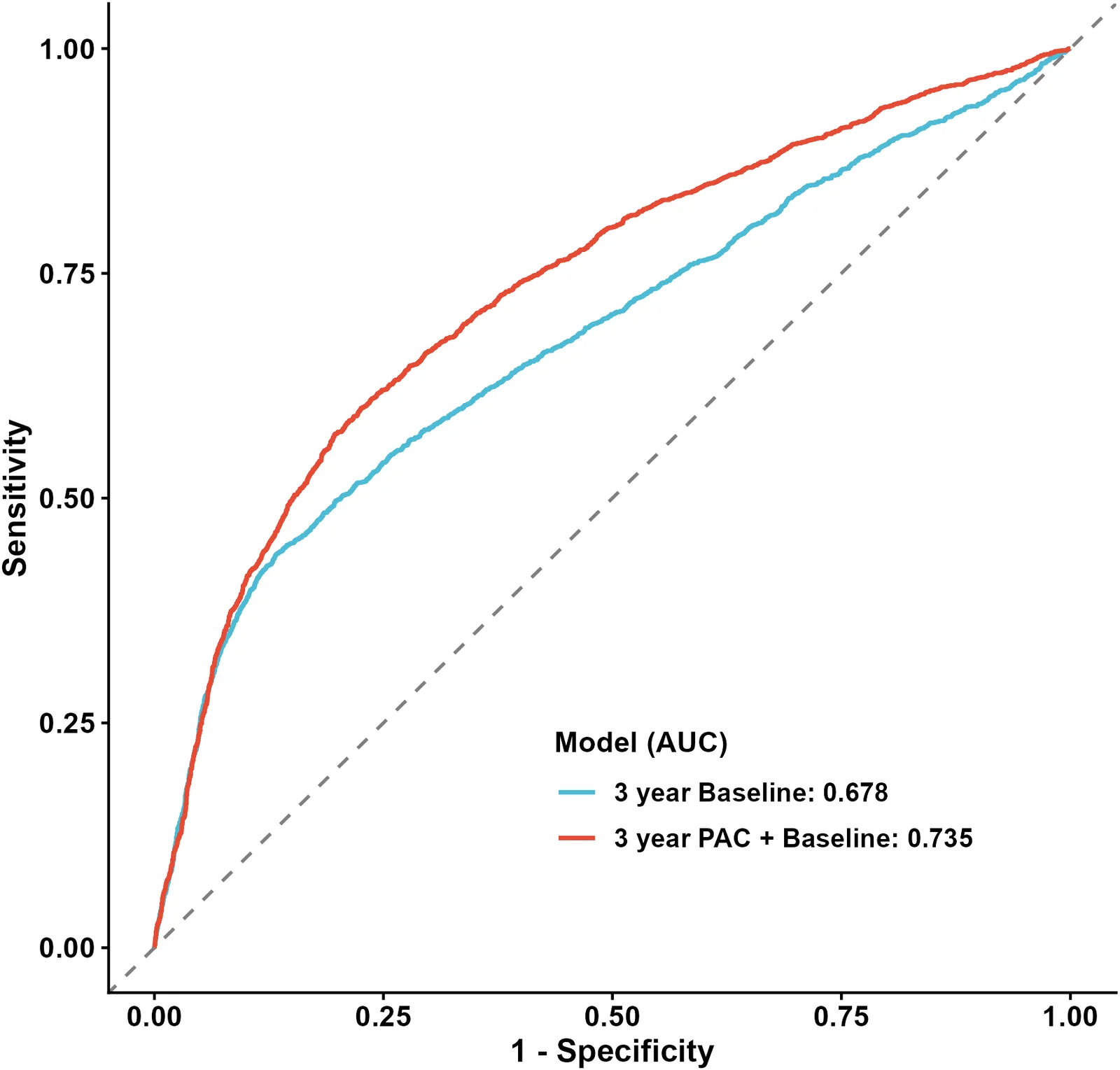
Time dependent ROC curves comparing the baseline model and the model incorporating PAC for 3 year prediction of CMM.

### 3.6 Decision curve analysis

Three-year risk DCA indicated that, across threshold probabilities of approximately 0–0.60, the model incorporating PAC with baseline predictors demonstrated consistently higher standardized net benefit compared with the baseline model alone (**Supplementary Figure S5**). Although the net benefit of both models gradually decreased and approached zero with increasing threshold probabilities, the PAC-integrated model maintained a higher estimated net benefit across most threshold ranges. These findings suggest that incorporating PAC into the prediction model may provide additional potential benefit for risk stratification of individuals at increased risk of CMM.

### 3.7 Calibration performance

Calibration plots demonstrated good agreement between predicted and observed 3-year CMM risks for both models. Compared with the baseline model, the PAC-integrated model showed a closer agreement between predicted and observed risks (**Supplementary Figure S6**).

### 3.8 Feature importance analysis

To further assess the relative importance of PAC in relation to incident CMM, we performed a Boruta feature-importance analysis based on a random forest algorithm. PAC showed greater importance than most of the other candidate variables (**Figure 7**), further supporting our findings that PAC may play a critical role in the development of CMM.

**Figure 7:**
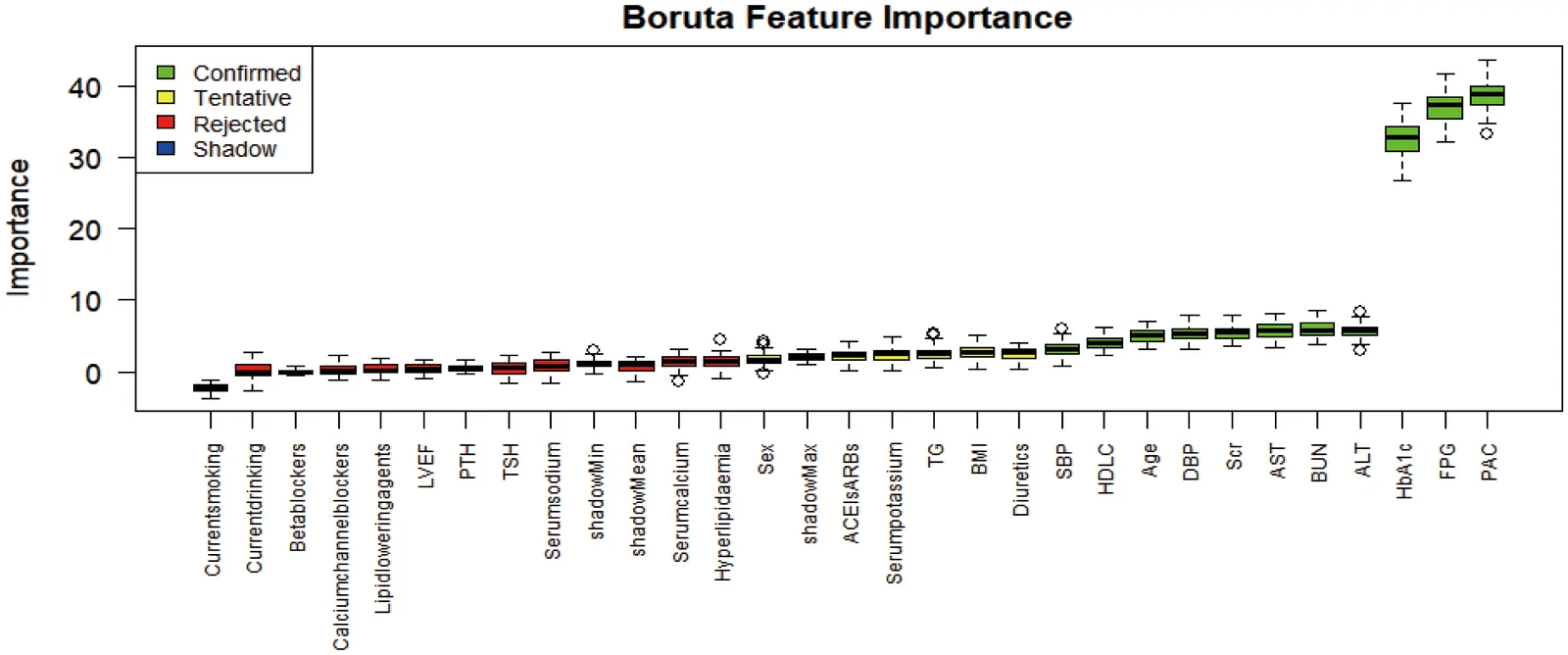
Feature importance ranking identified by the Boruta algorithm.

### 3.9 Sensitivity analyses

Multiple sensitivity analyses were performed to evaluate the robustness of the association between PAC and incident CMM. First, excluding participants with a follow-up duration of less than 1 year yielded results consistent with the primary analysis (**Supplementary Table S2**). Second, after excluding CMM events occurring during the early follow-up period (e.g., within the first 1–2 years) to minimize the potential influence of reverse causation, the association between PAC and CMM risk remained largely unchanged (**Supplementary Table S3**). Third, removal of extreme PAC values from the upper and lower 1% tails of the distribution did not materially alter the results (**Supplementary Table S4**). Fourth, exclusion of participants receiving diuretic therapy produced similar findings, further confirming the robustness of the observed association (**Supplementary Table S5**). Additionally, the E-value analysis suggested that an unmeasured confounder would require a relatively strong association with both PAC and CMM risk to fully explain the observed association, indicating that residual confounding was unlikely to account for the findings (**Supplementary Table S6**). Finally, because the number of deaths during follow-up was limited, the potential influence of competing risks on risk estimates was considered minimal. Therefore, competing risk regression models, such as the Fine–Gray subdistribution hazard model, were not additionally performed.

## 4 Discussion

In this retrospective cohort study, we investigated the association between baseline PAC levels and the risk of CMM. Our findings demonstrated that higher baseline PAC levels were significantly associated with an increased risk of incident CMM, even after comprehensive adjustment for potential confounding factors. Furthermore, RCS analysis revealed a significant nonlinear relationship between PAC and CMM risk (*P* for nonlinearity <0.001), with the risk curve showing an upward trend around a PAC level of 15.34 ng/dL. Notably, serum potassium levels may represent a potential modifier of the association between PAC and CMM risk. However, given the inconsistent findings between categorical and continuous interaction analyses, this observation should be interpreted cautiously and requires further validation. Moreover, incorporation of PAC into the established prediction model resulted in an approximately 6% improvement in the AUC and a continuous NRI of 0.372, suggesting that PAC provides additional incremental prognostic information beyond traditional risk factors.

These findings suggest that PAC may serve as a potential biomarker for further cardiometabolic risk stratification and suggest new opportunities for targeted prevention strategies among patients with hypertension.

PAC has traditionally been recognized as a key hormone involved in the regulation of blood volume, electrolyte homeostasis, and blood pressure control (22). However, accumulating evidence from experimental and clinical studies has demonstrated that PAC exerts broader pathological effects beyond hemodynamic regulation, contributing to multisystem organ injury (23, 24). Under pathological conditions, excessive activation of the mineralocorticoid receptor (MR) pathway by PAC promotes oxidative stress, inflammation, endothelial dysfunction, and fibrotic remodeling in cardiac and vascular tissues, thereby accelerating cardiovascular target-organ damage (25). Carrera-Chimal et al. demonstrated that MR antagonists exert cardioprotective effects across multiple disease models, including heart failure, myocardial infarction, atherosclerosis, and cardiorenal disease, further supporting the critical role of aberrant MR activation in the pathogenesis and progression of cardiovascular disorders (26). Beyond cardiovascular effects, accumulating evidence suggests that excessive MR activation may also contribute to metabolic abnormalities through mechanisms involving insulin resistance, adipose tissue inflammation, and oxidative stress, thereby facilitating the development of metabolic diseases such as diabetes mellitus (27, 28). These findings provide a biological rationale for the potential involvement of PAC signaling in the development of cardiometabolic multimorbidity, a condition characterized by the coexistence of multiple metabolic and cardiovascular disorders.

Importantly, Huang et al. reported that the global prevalence of primary aldosteronism (PA) was approximately 9.4%, and compared with patients with essential hypertension, individuals with PA exhibited substantially higher risks of coronary heart disease, stroke, heart failure, and atrial fibrillation, with odds ratios (ORs) of 1.88, 2.50, 2.06, and 3.17, respectively (29). Furthermore, Qian et al. demonstrated that medically treated patients with PA continued to experience a significantly higher risk of stroke than patients with essential hypertension (OR, 1.88; 95% CI, 1.68–2.11), whereas adrenalectomy markedly reduced stroke risk (OR, 0.57; 95% CI, 0.35–0.93), resulting in a risk level comparable to that observed in patients with essential hypertension (30). Collectively, these studies indicate that patients with PA remain at substantially elevated risk of cardiovascular events, particularly stroke, coronary heart disease, heart failure, and atrial fibrillation, even when blood pressure levels are comparable. Nevertheless, PA represents only the extreme end of the aldosterone excess spectrum. Increasing evidence suggests that modest elevations in PAC among individuals without meeting the diagnostic criteria for PA are also associated with target-organ damage and increased cardiovascular risk (31, 32). Extending previous findings, our study provides novel evidence from the perspective of CMM, demonstrating for the first time that continuously elevated PAC levels are associated with a dose–response increase in the risk of developing multiple coexisting cardiometabolic disorders. These results suggest that aldosterone-related pathophysiological activation may contribute to the broader cardiometabolic disease burden beyond clinically overt PA.

Our findings further suggest that serum potassium may modify the association between PAC and CMM. PAC and potassium homeostasis are closely linked through well-established physiological mechanisms. PAC promotes sodium reabsorption and potassium excretion in the distal nephron, and persistently elevated PAC levels may therefore be accompanied by hypokalemia (33). Nevertheless, accumulating evidence indicates that PAC-related tissue injury is not entirely dependent on the presence of hypokalemia. Mineralocorticoid receptor activation may also be associated with inflammation, oxidative stress, fibrosis, and cardiometabolic injury (23, 34, 35). In the subgroup analysis, each 1-standard-deviation increase in PAC was associated with a 23% higher risk of CMM among patients with serum potassium levels ≥3.5 mmol/L (HR, 1.23; 95% CI, 1.18–1.27). The corresponding estimate was weaker among those with serum potassium levels <3.5 mmol/L (HR, 1.09; 95% CI, 1.01–1.18), with a *P* value of 0.035 for interaction. From a clinically defined subgroup perspective, these findings indicate that the PAC–CMM association may differ according to potassium status. The bivariate penalized spline analysis provided additional evidence of a complex nonlinear pattern across combinations of PAC and serum potassium. At lower potassium levels, the estimated CMM risk was relatively elevated even when PAC was not markedly increased. In contrast, within the normokalemic range, higher PAC levels were associated with greater estimated CMM risk. This pattern may indicate that abnormal potassium status is not only a clinical correlate of PAC activity but also a marker of the broader physiological context in which PAC-related cardiometabolic abnormalities occur. Accordingly, screening for PAC abnormalities solely on the basis of hypokalemia may fail to identify some normokalemic patients with elevated PAC who nevertheless have a higher estimated risk of CMM. Joint consideration of PAC and serum potassium may therefore provide additional information for risk stratification, although the clinical value of such an approach requires further evaluation. However, when serum potassium was modeled as a continuous variable and its overall nonlinear interaction with PAC was assessed using flexible spline functions, the interaction did not reach statistical significance (P = 0.244). The different findings obtained from the categorical and continuous analyses may have several explanations. First, 3.5 mmol/L is a clinically established threshold for defining hypokalemia and is readily applicable in practice, but it may not represent the optimal biological or statistical cut point for variation in the PAC–CMM association. The interaction observed in the stratified analysis may therefore be partly dependent on the selected threshold. Second, the relatively small number of patients with serum potassium levels <3.5 mmol/L may have reduced the precision of the subgroup-specific estimates and limited the statistical power of the interaction analysis. Third, the two approaches address related but distinct questions. Analysis based on a single clinical threshold compares effect estimates on either side of that threshold, whereas a continuous nonlinear interaction model examines whether the association with PAC varies systematically across the entire potassium distribution. These approaches differ in their underlying assumptions, interpretation, and statistical efficiency. Taken together, serum potassium may represent a potentially relevant effect modifier of the association between PAC and CMM, but the nature of this variation remains uncertain. It is not yet clear whether the observed pattern reflects a threshold effect, a continuous nonlinear relationship, or differences related to other clinical characteristics. These findings should therefore be treated as exploratory and interpreted cautiously. Confirmation is needed in larger prospective cohorts with a greater proportion of patients with hypokalemia, as well as in mechanistic studies designed to clarify the underlying biological pathways. From a methodological perspective, our results also highlight the value of combining analyses based on prespecified clinical thresholds with continuous nonlinear interaction models. This complementary strategy may provide a more complete description of possible effect modification and reduce the likelihood of overinterpreting findings from any single analytical approach.

This study has several important strengths. First, we utilized a large-scale real-world hypertensive cohort with relatively long-term follow-up, enabling a comprehensive evaluation of the association between PAC levels and the long-term risk of CMM. Second, by incorporating restricted cubic spline analyses, threshold analyses, and bivariate spline modeling, this study not only confirmed the presence of an association but also characterized the nonlinear relationship and potential interaction patterns underlying the association between PAC and CMM risk. Third, we further evaluated the incremental predictive value of PAC beyond traditional risk factors, extending our findings from epidemiological associations to potential clinical prediction utility and enhancing their translational relevance. Nevertheless, several limitations should be acknowledged. First, because this was a single-center retrospective cohort study, the possibility of selection bias and residual confounding cannot be completely excluded. Future multicenter prospective studies are warranted to validate our findings. Second, PAC was measured only once at baseline, which may not fully capture longitudinal variations in aldosterone exposure over time. Future investigations incorporating repeated PAC measurements or cumulative exposure indicators are needed to better characterize the long-term effects of aldosterone. Third, although extensive statistical adjustments were performed, the observational design of this study precludes definitive conclusions regarding causality. Fourth, CMM was defined as the coexistence of diabetes mellitus, cardiovascular disease, and stroke; however, these individual disease components may involve distinct underlying pathophysiological pathways. Future studies should further investigate whether PAC differentially influences specific cardiometabolic outcomes. Fourth, antihypertensive medications may influence RAAS activity and consequently affect PAC, PRA, and serum potassium levels. Although medication management, multivariable adjustment, and medication-stratified analyses were performed to minimize this potential bias, residual effects of medication-induced alterations in the RAAS cannot be completely excluded. Furthermore, given the retrospective design of this study, not all participants underwent comprehensive screening for PA. Therefore, the possibility of undiagnosed or subclinical PA, as well as residual misclassification of PA status, cannot be entirely excluded and should be considered when interpreting the association between PAC and incident CMM. Finally, although the observed associations were statistically robust across multiple analyses, the retrospective and observational nature of the study limits causal inference. Therefore, our findings should be interpreted as hypothesis-generating evidence. The estimated PAC inflection point of 15.34 ng/dL identified by RCS analysis and the potential modifying effect of serum potassium require further validation in large-scale prospective cohorts and mechanistic studies.

## 5 Conclusion

In conclusion, higher PAC was associated with a greater incidence of CMM among individuals with hypertension, with a nonlinear pattern across PAC levels. The strength of this association differed according to serum potassium status. Adding PAC to a conventional prediction model also yielded incremental discrimination and greater net clinical benefit. PAC may therefore provide additional information for cardiometabolic risk stratification in hypertensive populations. However, its clinical applicability and the biological pathways underlying these associations warrant confirmation in prospective studies.

## Data Availability

The raw data supporting the conclusions of this article will be made available by the authors, without undue reservation.

## References

[1] Miranda JJ Barrientos-Gutiérrez T Corvalan C Hyder AA Lazo-Porras M Oni T . Understanding the rise of cardiometabolic diseases in low- and middle-income countries. Nat Med. (2019) 25:1667–79. doi: 10.1038/s41591-019-0644-7 31700182

[2] Su B Liu C Chen L Wu Y Li J Zheng X . Long-term exposure to PM2.5 and O3 with cardiometabolic multimorbidity: Evidence among Chinese elderly population from 462 cities. Ecotoxicol Environ Saf. (2023) 255:114790. doi: 10.1016/j.ecoenv.2023.114790 36948004

[3] Zhang Y Zhou Y Kivimäki M Holt-Lunstad J Carrillo-Larco RM Xu X . Cardiometabolic multimorbidity, social activity, and joint trajectories of physical disability, depressive symptom, and cognitive function in mid-to-late life: a multicohort study. BMC Med. (2025) 23:577. doi: 10.1186/s12916-025-04400-8 41126238 PMC12542226

[4] Di Angelantonio E Kaptoge S Wormser D Willeit P Butterworth AS Emerging Risk Factors Collaboration . Association of cardiometabolic multimorbidity with mortality. JAMA. (2015) 314:52–60. doi: 10.1001/jama.2015.7008 26151266 PMC4664176

[5] Song S Cai X Shen D Hu J Zhu Q Ma H . Relationship between cumulative exposure and time course of plasma aldosterone concentrations and chronic kidney disease in hypertensive patients: a real-world cohort study. Am J Nephrol. (2025) 56:641–56. doi: 10.1159/000545451 40159235

[6] Zhang Z Hong J Hu J Zhu Q Wu T Shen D . Plasma aldosterone concentration and valvular heart disease in hypertensive patients: a large cross-sectional study. Sci Rep. (2026) 16. doi: 10.1038/s41598-026-46943-7 41951729 PMC13219609

[7] Zhang Y Cai X Song S Hu J Zhou P Cai K . Association of plasma aldosterone concentration with peripheral artery disease in hypertensive patients: insights from a large cross-sectional analysis. Front Cardiovasc Med. (2025) 12:1549878. doi: 10.3389/fcvm.2025.1549878 40201786 PMC11977419

[8] Wang B Liu Z Bu T Rangarajan S Ma Q Li X . The interplay between antihypertensive medication and lifestyle with cardiovascular outcomes in patients with hypertension: the PURE-China study. EPMA J. (2026) 17:73–83. doi: 10.1007/s13167-026-00444-9 41822022 PMC12976333

[9] Fioretti F Testani JM Tio MC Pitt B Butler J . Aldosterone and aldosterone modulation in cardio-kidney diseases. J Am Coll Cardiol. (2025) 86:354–73. doi: 10.1016/j.jacc.2025.06.012 40738563

[10] Marney AM Brown NJ . Aldosterone and end-organ damage. Clin Sci (Lond). (2007) 113:267–78. doi: 10.1042/CS20070123 17683282

[11] Monticone S D'Ascenzo F Moretti C Williams TA Veglio F Gaita F . Cardiovascular events and target organ damage in primary aldosteronism compared with essential hypertension: a systematic review and meta-analysis. Lancet Diabetes Endocrinol. (2018) 6:41–50. doi: 10.1016/S2213-8587(17)30319-4 29129575

[12] Vaidya A Mulatero P Baudrand R Adler GK . The expanding spectrum of primary aldosteronism: implications for diagnosis, pathogenesis, and treatment. Endocr Rev. (2018) 39:1057–88. doi: 10.1210/er.2018-00139 30124805 PMC6260247

[13] Gan L Li N Heizhati M Wang H Yao L Cai L . Association between aldosterone and the risk of cardiovascular disease in hypertensive patients: a cohort study. Front Endocrinol (Lausanne). (2026) 17:1791569. doi: 10.3389/fendo.2026.1791569 42109741 PMC13149068

[14] Vasan RS Evans JC Larson MG Wilson PW Meigs JB Rifai N . Serum aldosterone and the incidence of hypertension in nonhypertensive persons. N Engl J Med. (2004) 351:33–41. doi: 10.1056/NEJMoa033263 15229305

[15] Tomaschitz A Pilz S Ritz E Meinitzer A Boehm BO März W . Plasma aldosterone levels are associated with increased cardiovascular mortality: the Ludwigshafen Risk and Cardiovascular Health (LURIC) study. Eur Heart J. (2010) 31:1237–47. doi: 10.1093/eurheartj/ehq019 20200015

[16] Brown JM Siddiqui M Calhoun DA Carey RM Hopkins PN Williams GH . The unrecognized prevalence of primary aldosteronism: a cross-sectional study. Ann Intern Med. (2020) 173:10–20. doi: 10.7326/M20-0065 32449886 PMC7459427

[17] Song S Cai X Hu J Zhu Q Shen D Ma H . Effectiveness of spironolactone in reducing osteoporosis and future fracture risk in middle-aged and elderly hypertensive patients. Drug Des Devel Ther. (2024) 18:2215–25. doi: 10.2147/DDDT.S466904 38882049 PMC11180452

[18] Song S Cai X Hu J Zhu Q Shen D Heizhati M . Correlation between plasma aldosterone concentration and bone mineral density in middle-aged and elderly hypertensive patients: potential impact on osteoporosis and future fracture risk. Front Endocrinol (Lausanne). (2024) 15:1373862. doi: 10.3389/fendo.2024.1373862 38808106 PMC11130431

[19] Dove A Guo J Marseglia A Fastbom J Vetrano DL Fratiglioni L . Cardiometabolic multimorbidity and incident dementia: the Swedish twin registry. Eur Heart J. (2023) 44:573–82. doi: 10.1093/eurheartj/ehac744 36577740 PMC9925275

[20] Xu Q Tian X Xia X Zhang Y Zheng M Wang A . Estimated glucose disposal rate, high sensitivity C-reactive protein and cardiometabolic multimorbidity in middle-aged and older Chinese adults: a nationwide prospective cohort study. Diabetes Res Clin Pract. (2024) 217:111894. doi: 10.1016/j.diabres.2024.111894 39414087

[21] Wan B Wang S Hu S Han W Qiu S Zhu L . The comprehensive effects of high-sensitivity C-reactive protein and triglyceride glucose index on cardiometabolic multimorbidity. Front Endocrinol (Lausanne). (2025) 16:1511319. doi: 10.3389/fendo.2025.1511319 40235659 PMC11996647

[22] Feldman RD Sanjanwala R Padwal R Leung AA . Revising the roles of aldosterone in vascular physiology and pathophysiology: from electocortin to baxdrostat. Can J Cardiol. (2023) 39:1808–15. doi: 10.1016/j.cjca.2023.08.035 37734710

[23] Brown JM . Adverse effects of aldosterone: beyond blood pressure. J Am Heart Assoc. (2024) 13:e030142. doi: 10.1161/JAHA.123.030142 38497438 PMC11179780

[24] Epstein M . Aldosterone and mineralocorticoid receptor signaling as determinants of cardiovascular and renal injury: from Hans Selye to the present. Am J Nephrol. (2021) 52:209–16. doi: 10.1159/000515622 33857953

[25] Ferreira NS Tostes RC Paradis P Schiffrin EL . Aldosterone, inflammation, immune system, and hypertension. Am J Hypertens. (2021) 34:15–27. doi: 10.1093/ajh/hpaa137 32820797 PMC7891246

[26] Barrera-Chimal J Bonnard B Jaisser F . Roles of mineralocorticoid receptors in cardiovascular and cardiorenal diseases. Annu Rev Physiol. (2022) 84:585–610. doi: 10.1146/annurev-physiol-060821-013950 35143332

[27] Jia G Lockette W Sowers JR . Mineralocorticoid receptors in the pathogenesis of insulin resistance and related disorders: from basic studies to clinical disease. Am J Physiol Regul Integr Comp Physiol. (2021) 320:R276–86. doi: 10.1152/ajpregu.00280.2020 33438511 PMC7988773

[28] Thuzar M Stowasser M . The mineralocorticoid receptor-an emerging player in metabolic syndrome. J Hum Hypertens. (2021) 35:117–23. doi: 10.1038/s41371-020-00467-3 33526798

[29] Huang M Li J Zhao X Fu R Li X Jiang W . Global and regional prevalence and cardiovascular risk of primary aldosteronism: a systematic review and meta-analysis. Curr Probl Cardiol. (2024) 49:102791. doi: 10.1016/j.cpcardiol.2024.102791 39127431

[30] Qian N Xu J Wang Y . Stroke risks in primary aldosteronism with different treatments: a systematic review and meta-analysis. J Cardiovasc Dev Dis. (2022) 9. doi: 10.3390/jcdd9090300 36135445 PMC9505464

[31] Cai X Song S Hu J Zhu Q Shen D Yang W . Association of the trajectory of plasma aldosterone concentration with the risk of cardiovascular disease in patients with hypertension: a cohort study. Sci Rep. (2024) 14:4906. doi: 10.1038/s41598-024-54971-4 38418472 PMC10902285

[32] Brown JM Wijkman MO Claggett BL Shah AM Ballantyne CM Coresh J . Cardiac structure and function across the spectrum of aldosteronism: the Atherosclerosis Risk in Communities Study. Hypertension. (2022) 79:1984–93. doi: 10.1161/HYPERTENSIONAHA.122.19134 35582954 PMC9759338

[33] Leipziger J Praetorius H . How does aldosterone work in the β-intercalated cell. J Am Soc Nephrol. (2020) 31:451–2. doi: 10.1681/ASN.2020010077 32065116 PMC7062213

[34] Savarese G Lindberg F Filippatos G Butler J Anker SD . Mineralocorticoid receptor overactivation: targeting systemic impact with non-steroidal mineralocorticoid receptor antagonists. Diabetologia. (2024) 67:246–62. doi: 10.1007/s00125-023-06031-1 38127122 PMC10789668

[35] McCurley A Jaffe IZ . Mineralocorticoid receptors in vascular function and disease. Mol Cell Endocrinol. (2012) 350:256–65. doi: 10.1016/j.mce.2011.06.014 21723914 PMC3214604

