# Supplementary material for: Dose–response association and predictive value of plasma aldosterone concentration for cardiometabolic multimorbidity in patients with hypertension: a retrospective cohort study
Source: Front Endocrinol (Lausanne). 2026 Oct 16;17:1965927. doi: 10.3389/fendo.2026.1965927 (PMC13719405; doi:10.3389/fendo.2026.1965927)
Supplement: Supplementary file 1 [file SupplementaryFile1.docx]

***Supplementary Material***

1 **Supplemental material and methods**

**Baseline examination**

Anthropometric measurements were taken by trained nurses. Data for height and weight were acquired following a protocol standardized to an accuracy of 0.1 kg and 0.1 cm, respectively. Current smokers were defined as having smoked 100 cigarettes in their lifetime and currently smoking. Blood pressure was measured using a mercury sphygmomanometer after the patient had rested quietly for at least 10 minutes, and the average of multiple measurements was taken as the systolic and diastolic blood pressure values. All biochemical tests were measured by blood sampling after an overnight fast.

**Definitions**

Criteria for hypertension included self-reported hypertension, current use of anti-hypertensive medication, or systolic blood pressure (SBP) ≥ 140 mmHg and/or diastolic blood pressure (DBP) ≥ 90 mmHg recorded for at least three consecutive readings. To identify primary aldosteronism (PA) according to the Endocrine Society's Clinical Practice Guidelines^[1]^. For plasma aldosterone concentration (PAC) measurements, participants were seated for 30 minutes after at least 2 hours of ambulatory activity, and blood samples were drawn between 8:00 AM and 11:00 AM. All patients received relevant preparatory instructions prior to aldosterone determination. For patients scheduled to undergo hormonal testing, it was recommended to discontinue interfering medications that could affect the renin–angiotensin–aldosterone system (RAAS) for 4–6 weeks before testing. For patients who could not discontinue these medications due to blood pressure control requirements, it was recommended to adjust the antihypertensive regimen to agents with less impact on plasma renin activity (PRA) and PAC 4–6 weeks before testing, including α-blockers and/or non-dihydropyridine calcium channel blockers (e.g., verapamil). For patients with severe hypertension or significant symptoms, hormonal testing was performed while continuing antihypertensive therapy, with priority given to agents that have minimal interference with PAC and PRA measurements. For patients with hypokalemia, serum potassium levels were corrected to near-normal range (approximately 3.9–4.0 mmol/L) through oral potassium supplementation before testing. All patients maintained normal dietary habits and adequate dietary salt intake (daily NaCl intake ≥6 g) prior to testing. PAC was measured using a radioimmunoassay method and quantitatively determined using the DSL-8600 Active Aldosterone Coated Tube Radioimmunoassay Kit (Diagnostic Systems Laboratories, Webster, TX, USA). The measurement of PRA was conducted using a commercial radioimmunoassay kit (Northern Bio-Technology Center, Beijing, China). The aldosterone‑to‑renin ratio (ARR) was calculated from PAC and PRA values.

Primary aldosteronism (PA) was diagnosed according to the clinical practice guidelines of the Endocrine Society. When PA was suspected—defined as PAC >12 ng/dL and an ARR ≥20—a saline infusion test (SIT) was further performed to confirm the diagnosis^[2]^. The diagnosis of PA followed the SIT criteria specified by the relevant Endocrine Society guidelines^[3]^. A post‑SIT PAC >10 ng/dL confirmed PA, whereas a post‑SIT PAC <5 ng/dL indicated a low likelihood of PA. Patients with a post‑SIT PAC between 5 and 10 ng/dL were classified as having an “indeterminate” diagnosis of PA. In such cases, the decision to diagnose or exclude PA was made by integrating clinical presentation, biochemical parameters (e.g., spontaneous or diuretic‑induced hypokalemia), and findings from adrenal thin‑slice CT scanning. It should be emphasised that the ARR was used only as a screening test and not as an independent diagnostic criterion. The definitive diagnosis of PA was based primarily on the SIT results, and in indeterminate cases, a comprehensive clinical evaluation was applied to reach a final judgment. The body mass index (BMI) was calculated as per the formula: weight (kg)/height^2^ (m). The primary study outcome was the incidence of CMM, defined as the coexistence of two or more cardiometabolic diseases (CMDs). Incident CMD cases were identified using the International Classification of Diseases, Tenth Revision (ICD-10) codes, combined with medical records and physician diagnoses. Diabetes mellitus was identified according to ICD-10 code E11. Stroke was identified using ICD-10 codes I60–I64 and I69. Heart disease was defined as clinically diagnosed cardiovascular conditions documented in the electronic medical records, including coronary heart disease, heart failure, and other cardiac diseases diagnosed by physicians based on relevant ICD-10 codes (I20–I25, I50, and other applicable cardiac disease codes). The onset date of CMM was defined as the date when the second cardiometabolic disease was first diagnosed during follow-up.

**Details of the statistical analyses**.

All baseline variables in this study were derived from electronic medical records and standardized questionnaire surveys. During the data cleaning phase, logical range checks were performed for continuous variables, and frequency checks were conducted for categorical variables to correct potential data entry errors. For missing data in each variable, multiple imputation was performed using the R package `mice` (Multivariate Imputation by Chained Equations). The imputation model included all analytical variables and outcome indicators, generating five complete datasets, which were subsequently pooled using Rubin's rules. In baseline characteristic analyses, continuous variables following a normal distribution were presented as mean (standard deviation) and compared between groups using one-way analysis of variance (ANOVA); continuous variables not following a normal distribution were presented as geometric means with 95% confidence intervals and compared using the Kruskal–Wallis test; categorical variables were presented as frequencies (percentages) and compared using the chi-square test or Fisher's exact test as appropriate. Normality was assessed using the Kolmogorov–Smirnov test. All continuous variables were evaluated for multicollinearity using the variance inflation factor (VIF) before inclusion in regression models, and variables with VIF ≥ 10 were excluded. The Kaplan–Meier method was used to estimate the cumulative incidence of CMM across different baseline PAC groups, with survival curves plotted accordingly. Between-group differences in cumulative incidence were compared using the log-rank test, and the follow-up endpoint was defined as the date of first CMM event occurrence or the last follow-up date. To evaluate the independent association between PAC and CMM risk, four hierarchically adjusted multivariable Cox proportional hazards regression models were constructed: Model 1 was unadjusted for covariates; Model 2 was adjusted for sex, age, BMI, current smoking, and current drinking status; Model 3 was further adjusted for systolic blood pressure, diastolic blood pressure, ALT, AST, triglycerides, HDL-C, LVEF, serum potassium, serum calcium, and serum sodium; Model 4 (fully adjusted model) additionally adjusted for serum creatinine and the use of antihypertensive medications including ACEIs/ARBs, calcium channel blockers, beta-blockers, and diuretics. The proportional hazards assumption for all Cox models was tested using scaled Schoenfeld residuals, and the global test P-values were all > 0.05, indicating that the PH assumption was not violated. Restricted cubic spline (RCS) models were employed to examine the dose–response relationship between PAC and CMM, with knots placed at the 10th, 50th, and 90th percentiles of the PAC distribution, and non-linear trends were evaluated using likelihood ratio tests. To explore potential effect modifiers, subgroup analyses were performed stratified by sex, age, BMI, smoking and drinking status, serum potassium (< 3.5 vs. ≥ 3.5 mmol/L), PRA, ARR, and history of hyperlipidemia. Multivariable Cox models were fitted within each subgroup to calculate HRs and 95% CIs, and interaction effects were assessed by including product terms in the Cox models and evaluating them with likelihood ratio tests. To further characterize the combined effect of serum potassium and PAC, RCS analyses stratified by serum potassium level and bivariate penalized spline models were applied to jointly estimate the smooth non-linear effects of these two variables on CMM risk, with penalty parameters selected via generalized cross-validation. The Boruta algorithm, a random forest-based wrapper feature selection method, was used to rank variable importance by iteratively comparing original variables with random shadow variables to identify true predictors significantly associated with the outcome. To assess the incremental predictive value of PAC for incident CMM, a baseline model including conventional risk factors was compared with an extended model incorporating PAC as a continuous variable. The baseline model consisted of age, sex, BMI, SBP, DBP, smoking and drinking status, TG, HDL-C, HbA1c, LVEF, FPG, CCB, beta-blockers, ACEI/ARB, and diuretics All prediction analyses were prespecified using a 3-year prediction horizon, which was selected based on the median follow-up duration of the cohort. Because incident CMM was analyzed as a time-to-event outcome with variable follow-up durations and right censoring, model discrimination was evaluated using time-dependent receiver operating characteristic (ROC) analysis with inverse probability of censoring weighting (IPCW). The area under the time-dependent ROC curve (AUC) and corresponding 95% confidence intervals (CIs) were calculated, and differences in discrimination between models were assessed.Internal validation was performed using 1,000 bootstrap resampling iterations to estimate optimism-corrected model performance. The incremental predictive value of PAC was further assessed using continuous survival-based net reclassification improvement (NRI) and integrated discrimination improvement (IDI), based on predicted 3-year absolute risks derived from Cox proportional hazards regression models while accounting for censoring. Model calibration was assessed by comparing predicted and observed 3-year CMM risks using calibration plots. Decision curve analysis (DCA) was performed using predicted 3-year risks to evaluate the potential clinical utility of adding PAC to the baseline model. Standardized net benefits of the baseline and PAC-integrated models were compared across a range of clinically relevant threshold probabilities. To ensure the robustness of our findings, several sensitivity analyses were conducted: (1) excluding participants with < 1 year of follow-up; (2) excluding CMM events occurring during the early follow-up period (first 1 and 2 years) to mitigate reverse causation; (3) removing extreme values at the upper and lower 1% tails of the PAC distribution; (4) excluding participants using diuretics at baseline; and (5) calculating E-values to assess the potential impact of unmeasured confounding on the observed results. Given the very low number of deaths during follow-up, the Fine–Gray competing risk model was not applied. All statistical analyses were performed using R software (version 4.5.3). All tests were two-sided, and a significance level of *P* < 0.05 was considered statistically significant.

**References**

[1]	Funder JW, Carey RM, Mantero F, et al. The Management of Primary Aldosteronism: Case Detection, Diagnosis, and Treatment: An Endocrine Society Clinical Practice Guideline. J Clin Endocrinol Metab. 2016. 101(5): 1889-916.

[2]	Maiturouzi M, Zhu Q, Zhang D, et al. Associations between thyroid function, thyroid diseases, and primary aldosteronism. Eur J Endocrinol. 2024. 191(2): 262-270.

[3]	Zhu Q, Heizhati M, Lin M, et al. Higher Plasma Aldosterone Concentrations Are Associated With Elevated Risk of Aortic Dissection and Aneurysm: a Case-Control Study. Hypertension. 2022. 79(4): 736-746.

2 **Supplementary Tables**

Table S1. Medication Stratification in Hypertensive Patients with PAC and CMM

| **CMM** | | | |
| --- | --- | --- | --- |
| PAC (per 1- SD increase) | P value | HR (95% CI) | P for interaction |
| Overall | <0.001 | 1.21 (1.17, 1.24) |  |
| Beta-blockers |  |  | 0.074 |
| No | <0.001 | 1.23 (1.18, 1.27) |  |
| Yes | <0.001 | 1.15 (1.09, 1.22) |  |
| CCB |  |  | 0.468 |
| No | <0.001 | 1.16 (1.07, 1.27) |  |
| Yes | <0.001 | 1.21 (1.17, 1.25) |  |
| ACEIs/ARBs |  |  | 0.243 |
| No | <0.001 | 1.07 (1.04, 1.11) |  |
| Yes | <0.001 | 1.16 (1.14, 1.19) |  |
| Diuretics |  |  | 0.748 |
| No | <0.001 | 1.21 (1.16, 1.25) |  |
| Yes | <0.001 | 1.19 (1.12, 1.28) |  |
| Lipidloweringagents |  |  | 0.830 |
| No | <0.001 | 1.20 (1.16, 1.25) |  |
| Yes | <0.001 | 1.21 (1.15, 1.28) |  |

Abbreviations: PAC, plasma aldosterone concentration; HR, Hazard Ratio; CI, Confidence Interval.

Other abbreviations, see Table 1.

Table S2. Sensitivity analysis excluding participants with follow-up 1 year

| Exposure | Model 1 | Model 2 | Model 3 | Model 4 | Bootstraping^a^ |
| --- | --- | --- | --- | --- | --- |
|  | HR (95% CI) P | HR (95% CI) P | HR (95% CI) P | HR (95% CI) P | HR (95% CI) P |
| CMM | | | | | |
| PAC (per 1-ng/dL increase) | 1.02 (1.02, 1.03)  <0.001 | 1.02 (1.02, 1.03)  <0.001 | 1.02 (1.02, 1.03)  <0.001 | 1.02 (1.02, 1.03)  <0.001 | 1.02 (1.02, 1.03)  <0.001 |
| PAC (per 1 SD increase) | 1.15 (1.11, 1.19)  <0.001 | 1.17 (1.13, 1.21 )  <0.001 | 1.17 (1.13, 1.22)  <0.001 | 1.17 (1.13, 1.21)  <0.001 | 1.24 (1.19–1.29)  <0.001 |
| Quartiles of PAC |  |  |  |  |  |
| Q1 | Reference | Reference | Reference | Reference | Reference |
| Q2 | 1.16 (1.01, 1.33)  <0.001 | 1.18 (1.02, 1.35)  <0.001 | 1.17 (1.02, 1.35)  <0.001 | 1.18 (1.03, 1.36)  <0.001 | 1.18 (1.02, 1.38)  <0.001 |
|  |  |  |  |  |  |
| Q3 | 1.27 (1.11, 1.45) <0.001 | 1.33 (1.16, 1.52)  <0.001 | 1.33 (1.16, 1.52)  <0.001 | 1.31 (1.14, 1.50)  <0.001 | 1.31 (1.14, 1.51) <0.001 |
|  |  |  |  |  |  |
| Q4 | 1.56 (1.37, 1.77)  <0.001 | 1.66 (1.46, 1.88)  <0.001 | 1.65 (1.45, 1.87)  <0.001 | 1.65 (1.45, 1.87)  <0.001 | 1.65 (1.45, 1.89)  <0.001 |
| Q1-Q2 | Reference | Reference | Reference | Reference | Reference |
| Q3-Q4 | 1.32 (1.21, 1.45)  <0.001 | 1.38 (1.26, 1.51)  <0.001 | 1.38 (1.26, 1.52)  <0.001 | 1.36 (1.24, 1.50)  <0.001 | 1.37 (1.25, 1.52)  <0.001 |
| P for trend | <0.001 | <0.001 | <0.001 | <0.001 | <0.001 |

Notes:Model 1: No covariates were adjusted.

Model 2: Adjusted for SEX, AGE, BMI, Current smoking, Current drinking.

Model 3: Adjusted for SEX, AGE, BMI, Current smoking, Current drinking, SBP, DBP, ALT, AST, TG, HDL-C, LVEF, Serum potassium, Serum calcium, Serum sodium.

Model 4: Adjusted for SEX, AGE, BMI, Current smoking, Current drinking, SBP, DBP, ALT, AST, TG, HDL-C, LVEF, Serum potassium, Serum calcium, Serum sodium, Scr, ACEIs/ARBs; Calcium channel blockers, Beta-blockers, Diuretics.

^a^Bootstrapping was conducted with 1000 bootstrap samples via Adjusted Model 4

Abbreviations: ACEIs, angiotensin-converting enzyme inhibitors; ARBs, angiotensin receptor blockers; PAC, Plasma Aldosterone Concentration; HR, Hazard Ratio; CI, Confidence Interval; P, Probability.

Other abbreviations, see Table 1.

Table S3. Sensitivity analysis excluding participants who developed CMM within the first 2 years.

| Exposure | Model 1 | Model 2 | Model 3 | Model 4 | Bootstraping**^a^** |
| --- | --- | --- | --- | --- | --- |
|  | HR (95% CI) P | HR (95% CI) P | HR (95% CI) P | HR (95% CI) P | HR (95% CI) P |
| CMM | | | | | |
| PAC (per 1-ng/dL increase) | 1.02 (1.02, 1.03)  <0.001 | 1.02 (1.02, 1.03)  <0.001 | 1.02 (1.02, 1.03)  <0.001 | 1.02 (1.02, 1.03)  <0.001 | 1.02 (1.02, 1.03)  <0.001 |
| PAC (per 1 SD increase) | 1.19 (1.15–1.23)  <0.001 | 1.20 (1.17–1.24)  <0.001 | 1.21 (1.17–1.25  <0.001 | 1.22 (1.17–1.27)  <0.001 | 1.23 (1.18, 1.30)  <0.001 |
| Quartiles of PAC |  |  |  |  |  |
| Q1 | Reference | Reference | Reference | Reference | Reference |
| Q2 | 1.19 (1.02, 1.38)  <0.001 | 1.21 (1.04, 1.40)  <0.001 | 1.20 (1.04–1.40)  <0.001 | 1.22 (1.05, 1.42)  <0.001 | 1.22 (1.04, 1.44)  <0.001 |
|  |  |  |  |  |  |
| Q3 | 1.29 (1.12, 1.50)  <0.001 | 1.35 (1.17, 1.56)  <0.001 | 1.35 (1.16, 1.56)  <0.001 | 1.34 (1.16, 1.56)  <0.001 | 1.34 (1.15, 1.57)  <0.001 |
|  |  |  |  |  |  |
| Q4 | 1.62 (1.41, 1.85)  <0.001 | 1.72 (1.50–1.97)  <0.001 | 1.71 (1.49, 1.96)  <0.001 | 1.72 (1.50, 1.98)  <0.001 | 1.90 (1.61, 2.24)  <0.001 |
| Q1-Q2 | Reference | Reference | Reference | Reference | Reference |
| Q3-Q4 | 1.34 (1.22, 1.48)  <0.001 | 1.40 (1.27, 1.55)  <0.001 | 1.40 (1.27, 1.55)  <0.001 | 1.39 (1.26, 1.54)  <0.001 | 1.72 (1.49, 2.00)  <0.001 |
| P for trend | <0.001 | <0.001 | <0.001 | <0.001 | <0.001 |

Notes:Model 1: No covariates were adjusted.

Model 2: Adjusted for SEX, AGE, BMI, Current smoking, Current drinking.

Model 3: Adjusted for SEX, AGE, BMI, Current smoking, Current drinking, SBP, DBP, ALT, AST, TG, HDL-C, LVEF, Serum potassium, Serum calcium, Serum sodium.

Model 4: Adjusted for SEX, AGE, BMI, Current smoking, Current drinking, SBP, DBP, ALT, AST, TG, HDL-C, LVEF, Serum potassium, Serum calcium, Serum sodium, Scr, ACEIs/ARBs; Calcium channel blockers, Beta-blockers, Diuretics.

^a^Bootstrapping was conducted with 1000 bootstrap samples via Adjusted Model 4

Abbreviations: ACEIs, angiotensin-converting enzyme inhibitors; ARBs, angiotensin receptor blockers; PAC, Plasma Aldosterone Concentration; HR, Hazard Ratio; CI, Confidence Interval; P, Probability.

Other abbreviations, see Table 1.

Table S4. Sensitivity analysis after exclusion of outliers

| Exposure | Model 1 | Model 2 | Model 3 | Model 4 | Bootstrapinga |
| --- | --- | --- | --- | --- | --- |
|  | HR (95% CI) P | HR (95% CI) P | HR (95% CI) P | HR (95% CI) P | HR (95% CI) P |
| CMM | | | | | |
| PAC (per 1-ng/dL increase) | 1.03 (1.03, 1.04)  <0.001 | 1.04 (1.03, 1.04)  <0.001 | 1.04 (1.03, 1.04)  <0.001 | 1.04 (1.03, 1.04)  <0.001 | 1.04 (1.03, 1.04)  <0.001 |
| PAC (per 1 SD increase) | 1.27 (1.22, 1.32)  <0.001 | 1.30 (1.25, 1.35)  <0.001 | 1.30 (1.25, 1.36)  <0.001 | 1.30 (1.25, 1.36)  <0.001 | 1.30 (1.26, 1.36)  <0.001 |
| Quartiles of PAC |  |  |  |  |  |
| Q1 | Reference | Reference | Reference | Reference | Reference |
| Q2 | 1.13 (0.99, 1.30)  0.07 | 1.15 (1.00, 1.32)  0.04 | 1.13 (0.98, 1.30)  0.05 | 1.16 (1.01, 1.34)  <0.001 | 1.16 (1.01, 1.35)  <0.001 |
|  |  |  |  |  |  |
| Q3 | 1.22 (1.07, 1.40)  <0.001 | 1.28 (1.12, 1.47)  <0.001 | 1.28 (1.11, 1.47)  <0.001 | 1.28 (1.11, 1.47)  <0.001 | 1.28 (1.11, 1.47)  <0.001 |
|  |  |  |  |  |  |
| Q4 | 1.53 (1.34, 1.74)  <0.001 | 1.63 (1.43, 1.85)  <0.001 | 1.61 (1.41, 1.83)  <0.001 | 1.61 (1.41, 1.83)  <0.001 | 1.63 (1.44, 1.87)  <0.001 |
| Q1-Q2 | Reference | Reference | Reference | Reference | Reference |
| Q3-Q4 | 1.30 (1.18, 1.42)  <0.001 | 1.36 (1.24, 1.49)  <0.001 | 1.36 (1.24, 1.49)  <0.001 | 1.35 (1.23, 1.48)  <0.001 | 1.36 (1.24, 1.49)  <0.001 |
| P for trend | <0.001 | <0.001 | <0.001 | <0.001 | <0.001 |

Notes:Model 1: No covariates were adjusted.

Model 2: Adjusted for SEX, AGE, BMI, Current smoking, Current drinking.

Model 3: Adjusted for SEX, AGE, BMI, Current smoking, Current drinking, SBP, DBP, ALT, AST, TG, HDL-C, LVEF, Serum potassium, Serum calcium, Serum sodium.

Model 4: Adjusted for SEX, AGE, BMI, Current smoking, Current drinking, SBP, DBP, ALT, AST, TG, HDL-C, LVEF, Serum potassium, Serum calcium, Serum sodium, Scr, ACEIs/ARBs; Calcium channel blockers, Beta-blockers, Diuretics.

^a^Bootstrapping was conducted with 1000 bootstrap samples via Adjusted Model 4

Abbreviations: ACEIs, angiotensin-converting enzyme inhibitors; ARBs, angiotensin receptor blockers; PAC, Plasma Aldosterone Concentration; HR, Hazard Ratio; CI, Confidence Interval; P, Probability.

Other abbreviations, see Table 1.

Table S5. Sensitivity analysis excluding diuretic users

| Exposure | Model 1 | Model 2 | Model 3 | Model 4 | Bootstraping**^a^** |
| --- | --- | --- | --- | --- | --- |
|  | HR (95% CI) P | HR (95% CI) P | HR (95% CI) P | HR (95% CI) P | HR (95% CI) P |
| CMM | | | | | |
| PAC (per 1-ng/dL increase) | 1.02 (1.02, 1.03)  <0.001 | 1.02 (1.02, 1.03)  <0.001 | 1.02 (1.02, 1.03)  <0.001 | 1.03 (1.02, 1.03)  <0.001 | 1.03 (1.02, 1.03)  <0.001 |
| PAC (per 1 SD increase) | 1.20 (1.16, 1.25)  <0.001 | 1.22 (1.18, 1.26)  <0.001 | 1.22 (1.18, 1.27)  <0.001 | 1.23 (1.19, 1.27)  <0.001 | 1.23 (1.18, 1.29)  <0.001 |
| Quartiles of PAC |  |  |  |  |  |
| Q1 | Reference | Reference | Reference | Reference | Reference |
| Q2 | 1.26 (1.08, 1.47)  <0.001 | 1.28 (1.10, 1.50)  <0.001 | 1.29 (1.11, 1.50)  <0.001 | 1.30 (1.11, 1.51)  <0.001 | 1.30 (1.11, 1.51)  <0.001 |
|  |  |  |  |  |  |
| Q3 | 1.30 (1.12, 1.50)  <0.001 | 1.34 (1.15, 1.55)  <0.001 | 1.34 (1.15, 1.56)  <0.001 | 1.33 (1.14, 1.55)  <0.001 | 1.33 (1.14, 1.56)  <0.001 |
|  |  |  |  |  |  |
| Q4 | 1.68 (1.46, 1.93)  <0.001 | 1.77 (1.54, 2.04)  <0.001 | 1.75 (1.52, 2.02)  <0.001 | 1.77 (1.53, 2.04)  <0.001 | 1.77 (1.54, 2.05)  <0.001 |
| Q1-Q2 | Reference | Reference | Reference | Reference | Reference |
| Q3-Q4 | 1.33 (1.20, 1.47)  <0.001 | 1.37 (1.24, 1.52)  <0.001 | 1.37 (1.24, 1.51)  <0.001 | 1.36 (1.23, 1.50)  <0.001 | 1.37 (1.24, 1.52)  <0.001 |
| P for trend | <0.001 | <0.001 | <0.001 | <0.001 | <0.001 |

Notes:Model 1: No covariates were adjusted.

Model 2: Adjusted for SEX, AGE, BMI, Current smoking, Current drinking.

Model 3: Adjusted for SEX, AGE, BMI, Current smoking, Current drinking, SBP, DBP, ALT, AST, TG, HDL-C, LVEF, Serum potassium, Serum calcium, Serum sodium.

Model 4: Adjusted for SEX, AGE, BMI, Current smoking, Current drinking, SBP, DBP, ALT, AST, TG, HDL-C, LVEF, Serum potassium, Serum calcium, Serum sodium, Scr, ACEIs/ARBs; Calcium channel blockers, Beta-blockers, Diuretics.

^a^Bootstrapping was conducted with 1000 bootstrap samples via Adjusted Model 4

Abbreviations: ACEIs, angiotensin-converting enzyme inhibitors; ARBs, angiotensin receptor blockers; PAC, Plasma Aldosterone Concentration; HR, Hazard Ratio; CI, Confidence Interval; P, Probability.

Other abbreviations, see Table 1.

Table S6 .E-values for the Observed Associations Between PAC and CMM

|  | Model 1 | Model 2 | Model 3 | Model 4 | Model 5 | Bootstraping**^a^** |
| --- | --- | --- | --- | --- | --- | --- |
| CMM |  |  |  |  |  |  |
| PAC (per 1-ng/dL increase) | 1.02 (1.02, 1.03) | 1.02 (1.02, 1.03) | 1.02 (1.02, 1.03) | 1.02 (1.02, 1.03) | 1.03 (1.02, 1.03) | 1.02 (1.02, 1.03) |
| E-value for point estimate | 1.16 | 1.16 | 1.16 | 1.16 | 1.19 | 1.16 |
| PAC (per 1 SD increase) | 1.21 (1.17, 1.24) | 1.22 (1.19, 1.26) | 1.22 (1.19, 1.26) | 1.22 (1.18–1.26) | 1.23 (1.19, 1.29) | 1.21 (1.17, 1.24) |
| E-value for point estimate | 1.71 | 1.75 | 1.75 | 1.75 | 1.78 | 1.71 |

Notes:Model 1: No covariates were adjusted.

Model 2: Adjusted for SEX, AGE, BMI, Current smoking, Current drinking.

Model 3: Adjusted for SEX, AGE, BMI, Current smoking, Current drinking, SBP, DBP, ALT, AST, TG, HDL-C, LVEF, Serum potassium, Serum calcium, Serum sodium.

Model 4: Adjusted for SEX, AGE, BMI, Current smoking, Current drinking, SBP, DBP, ALT, AST, TG, HDL-C, LVEF, Serum potassium, Serum calcium, Serum sodium, Scr, ACEIs/ARBs; Calcium channel blockers, Beta-blockers, Diuretics.

^a^Bootstrapping was conducted with 1000 bootstrap samples via Adjusted Model 4

Other abbreviations, see Table 1.

**2.2	Supplementary Figures.**

Figure S1. Lollipop chart showing the missing rates of variables included in the analysis

Figure S2. Comparison of variance inflation factor (VIF) for each variable before and after variable filtering

Figure S3. Plot of Schoenfeld residuals against time in the Cox regression model CMM

Figure S4. Bivariate penalized spline model for the interaction between serum potassium and PAC on the relative risk of CMM

Figure S5. Decision curve analysis for the baseline model and the model incorporating PAC

Figure S6. Calibration plots of the baseline model and the model incorporating PAC for 3-year risk prediction of cardiometabolic multimorbidity
